# Supplementary material for: Longitudinal study of use and cost of subacromial decompression surgery: the need for effective evaluation of surgical procedures to prevent overtreatment and wasted resources
Source: BMJ Open. 2019 Aug 28;9(8):e030229. doi: 10.1136/bmjopen-2019-030229 (PMC6719759; doi:10.1136/bmjopen-2019-030229)
Supplement: Supplementary data [file bmjopen-2019-030229supp001.pdf]

APPENDIX A: METHODOLOGICAL DETAILS

Estimating Procedure Rates

National trends over time were estimated using directly standardised procedure rates[1] (per 100,000 population), with the population of England in 2016 as our standard population. We first summed the number of shoulder procedures, grouped by sex, quintiles of age, and financial year. These procedure counts were used to calculate annual age-sex-specific rates, by dividing by the appropriate age-sex-specific mid-year populations of England[2] (e.g. for the 2012/13 financial year, the mid-2012 populations were used). We weighted the annual age-sex-specific rates according to the population distribution of England in 2016, to produce directly standardised rates for each year. The standardised rates for 2016/17 are the same as the crude rates.

For comparison of smaller areas, we estimated indirectly standardised rates[3] per 100,000 population. We first calculated age-sex-specific rates for England in 2016/17, then multiplied these rates by the age-sex-specific population for the area of interest[2 4 5] (e.g. CCG) and summed the results. This produced the expected number of patients and procedures for that area, if it were to have the same age-sex-specific rates as England. The expected number was then compared to the observed number of patients and procedures for that area. A Poisson regression model was fitted to the observed counts for each year, with the expected counts as an offset and socio-economic deprivation (using the overall score from the English Indices of Multiple Deprivation[6]) and ethnicity (% white British[7]) as predictive factors. The model was then used to predict new expected counts for each area based on deprivation and ethnicity, and form indirectly standardised procedure ratios (observed / expected).

Estimating Procedure Costs

Costs were estimated for each financial year by linking HRG codes for each admission in HES with the Department of Health Payment by Results National Tariffs for the appropriate financial year.[8-17] Enhanced Tariff Option (ETO) tariffs were applied for 2015/16 as, following a dispute, 88% of providers agreed to use ETO tariffs for that financial year.[18] The National Tariffs provide costs for day cases and longer stays, for both elective and non-elective admissions. They also provide additional daily costs for admissions that go above a threshold number of days (termed excess bed days), which varies for different types of admission. To calculate the cost of admission, we excluded admissions without a discharge date (used to calculate number of bed days) or without a HRG code that matched to the National Tariffs (0.7% excluded). We then applied the relevant national tariff or alternatively the best practice tariff where applicable (only for HRG code HB62C under specified circumstances) and added excess bed day costs (if there were any). Following this, the special service top-up for orthopaedic procedures was applied for each year.

References

1. Standardization: a classic epidemiological method for the comparison of rates. *Epidemiol Bull* 2002;23(3):9-12. [published Online First: 2003/03/01]
2. Office for National Statistics. Lower Super Output Area Mid-Year Population Estimates [Available from: https://www.ons.gov.uk/peoplepopulationandcommunity/populationandmigration/populationestimates/datasets/lowersuperoutputareamidyearpopulationestimates accessed 30 November 2017.
3. Anon. Standardization: a classic epidemiological method for the comparison of rates. *Epidemiological Bulletin* 2002;23(3):9-12.
4. Office for National Statistics. Lower Layer Super Output Area (2011) to Clinical Commissioning Group to Local Authority District (July 2015) Lookup in England: Office for National Statistics; [Available from: http://ons.maps.arcgis.com/home/item.html?id=7de88cd2369d4006925de49e8f3f2e272016.
5. Office for National Statistics. Output Area to Lower Layer Super Output Area to Middle Layer Super Output Area to Local Authority District (December 2011) Lookup in England and Wales [Available from: http://ons.maps.arcgis.com/home/item.html?id=baf6566ca08949c6bbd61ff81d9526da2016.

6. Government DoCaL. English Indices of Deprivation 2015 [Available from: https://www.gov.uk/government/statistics/english-indices-of-deprivation-20152016.
7. Nomis; Office for National Statistics. Ethnic group of the usual resident population of England and Wales as at census day, 27 March 2011 [Available from: https://www.nomisweb.co.uk/census/2011/ks201ew2016.
8. Department of Health. Payment by Results National Tariffs 2016/17 2016 [Available from: https://www.gov.uk/government/publications/nhs-national-tariff-payment-system-201617 accessed 30 November 2017.
9. Department of Health. Payment by Results Enhanced Tariff Option 2015/16 2015 [Available from: http://webarchive.nationalarchives.gov.uk/20161103233943/https://www.england.nhs.uk/resources/pay-syst/tariff-guide/ accessed 30 November 2017.
10. Department of Health. Payment by Results National Tariffs 2014/15 2014 [Available from: https://www.gov.uk/government/publications/national-tariff-payment-system-2014-to-2015 accessed 30 November 2017.
11. Department of Health. Payment by Results National Tariffs 2013/14 2013 [Available from: https://www.gov.uk/government/publications/payment-by-results-pbr-operational-guidance-and-tariffs accessed 30 November 2017.
12. Department of Health. Payment by Results National Tariffs 2012/13 2012 [Available from: https://www.gov.uk/government/publications/confirmation-of-payment-by-results-pbr-arrangements-for-2012-13 accessed 30 November 2017.
13. Department of Health. Payment by Results National Tariffs 2011/12 2011 [Available from: http://webarchive.nationalarchives.gov.uk/20130507170152/https://www.gov.uk/government/publications/confirmation-of-payment-by-results-pbr-arrangements- for-2011-12 accessed 30 November 2017.
14. Department of Health. Payment by Results National Tariffs 2010/11 2010 [Available from: https://data.gov.uk/dataset/payment-by-results-2010-11-national-tariff-information accessed 30 November 2017.
15. Department of Health. Payment by Results National Tariffs 2009/10 2009 [Available from: http://webarchive.nationalarchives.gov.uk/+/http://www.dh.gov.uk/en/Publicationsandstatistics/Publications/PublicationsPolicyAndGuidance/DH_094091 accessed 30 November 2017.
16. Department of Health. Payment by Results National Tariffs 2008/09 2008 [Available from: http://webarchive.nationalarchives.gov.uk/+/http://www.dh.gov.uk/en/Managingyourorganisation/Financeandplanning/NHSFinancialReforms/DH_081238 accessed 30 November 2017.
17. Department of Health. Payment by Results National Tariffs 2007/08 2007 [Available from: http://webarchive.nationalarchives.gov.uk/+/http://www.dh.gov.uk/en/Publicationsandstatistics/Publications/PublicationsPolicyAndGuidance/DH_062914 accessed 30 November 2017.
18. Grasic K, Mason AR, Street A. Paying for the quantity and quality of hospital care: the foundations and evolution of payment policy in England. *Health Econ Rev* 2015;5 doi: 10.1186/s13561-015-0050-x
