# Supplementary material for: Longitudinal study of use and cost of subacromial decompression surgery: the need for effective evaluation of surgical procedures to prevent overtreatment and wasted resources
Source: BMJ Open. 2019 Aug 28;9(8):e030229. doi: 10.1136/bmjopen-2019-030229 (PMC6719759; doi:10.1136/bmjopen-2019-030229)
Supplement: Author's manuscript [file bmjopen-2019-030229.draft_revisions.pdf]

BMJ Open

BMJ Open is committed to open peer review. As part of this commitment we make the peer review history of every article we publish publicly available.

When an article is published we post the peer reviewers' comments and the authors' responses online. We also post the versions of the paper that were used during peer review. These are the versions that the peer review comments apply to.

The versions of the paper that follow are the versions that were submitted during the peer review process. They are not the versions of record or the final published versions. They should not be cited or distributed as the published version of this manuscript.

BMJ Open is an open access journal and the full, final, typeset and author-corrected version of record of the manuscript is available on our site with no access controls, subscription charges or pay-per-view fees (http://bmjopen.bmj.com).

If you have any questions on BMJ Open's open peer review process please email info.bmjopen@bmj.com

BMJ Open

A longitudinal study of use and cost of subacromial decompression surgery: the need for effective evaluation of surgical procedures to prevent overtreatment and wasted resources.

Journal:	*BMJ Open*
Manuscript ID	bmjopen-2019-030229
Article Type:	Research
Date Submitted by the Author:	05-Mar-2019
Complete List of Authors:	Jones, Tim; University of Bristol, NIHR CLAHRC West<br>Carr, Andrew; University of Oxford, Nuffield Department of Orthopaedics, Rheumatology and Musculoskeletal Sciences<br>Beard, David; University of Oxford, Nuffield Department of Orthopaedics, Rheumatology and Musculoskeletal Sciences<br>Linton, Myles-Jay; University of Bristol, NIHR CLAHRC West<br>Rooshenas, Leila; University of Bristol, Population Health Sciences<br>Donovan, Jenny; University of Bristol, NIHR CLAHRC West<br>Hollingworth, William; University of Bristol, Population Health Sciences
Keywords:	subacromial decompression, shoulder surgery, England, commissioning, arthroscopy

SCHOLARONE™
Manuscripts

Title: A longitudinal study of use and cost of subacromial decompression surgery: the need for effective evaluation of surgical procedures to prevent overtreatment and wasted resources.

Authors: Tim Jones[1,2], Andrew Carr[3], David Beard[3], Myles-Jay Linton[1,2], Leila Rooshenas[2], Jenny Donovan[1,2], and William Hollingworth[1,2]

[1] NIHR CLAHRC West, University Hospitals Bristol NHS Foundation Trust, Bristol, BS1 2NT, UK

[2] Department of Population Health Sciences, Bristol Medical School, University of Bristol, BS8 2PS, UK

[3] Nuffield Department of Orthopaedics, Rheumatology, and Musculoskeletal Sciences (NDORMS), NIHR Biomedical Research Centre, Botnar Research Centre, University of Oxford, Oxford, UK

Corresponding author:

Tim Jones
Senior Research Associate
NIHR CLAHRC West,
9th Floor Whitefriars,
Lewin's Mead
Bristol, BS1 2NT
United Kingdom

Tel: ++44 (0)117 342 1265
Timothy.Jones@bristol.ac.uk

Word count [3,065]

Keywords:
Subacromial decompression; arthroscopy; shoulder surgery; England; commissioning

For peer review only

ABSTRACT

Objectives: To illustrate the need for better evaluation of surgical procedures, we investigated the use and cost of subacromial decompression in England over the last decade compared with other countries and explored how this related to the conduct and outcomes of randomised, placebo-controlled clinical trials.

Design: Longitudinal observational study using Hospital Episode Statistics linked to Payment by Results tariffs in England, 2007/8-2016/17.

Setting: Hospital care in England, Finland, New York State USA, Florida State USA, and Western Australia.

Participants: Patients with subacromial shoulder pain.

Interventions: Subacromial decompression.

Main outcome measures: National procedure rates, costs, and variation between clinical commissioning groups (CCGs) in England.

Results: Without robust clinical evidence, the use of subacromial decompression in England increased by 91% from 15,112 procedures (30 per 100,000 population) in 2007/8, to 28,802 procedures (52 per 100,000 population) in 2016/17, costing over £125 million per year. Rates of use of subacromial decompression are even higher internationally: Finland (131 per 100,000 in 2011), Florida State (130 per 100,000 in 2007), Western Australia (115 per 100,000 in 2013), and New York State (102 per 100,000 in 2006). Two randomised trials have recently (2018) shown the procedure to be no more effective than placebo or conservative approaches. Health systems appear unable to avoid the rapid widespread use of procedures of unknown effectiveness, and methods for ceasing ineffective treatments are under-developed.

Conclusions: Without good evidence, nearly 30,000 subacromial decompression procedures have been commissioned each year in England, costing over £1 billion since 2007/8. Even higher rates of procedures are carried out in countries with less regulated health systems. Randomised trials need to be initiated before widespread adoption of promising operative procedures to avoid over-

For peer review only - http://bmjopen.bmj.com/site/about/guidelines.xhtml

treatment and wasted resources, and methods to prevent or desist the use of ineffective procedures need to be expedited.

For peer review only

For peer review only - http://bmjopen.bmj.com/site/about/guidelines.xhtml

ARTICLE SUMMARY

Strengths and Limitations of this Study

- Our study used a national, longitudinal dataset over a 10-year period covering all NHS secondary care providers in England, and private provision for NHS-funded patients.
- Hospital Episode Statistics are linked to hospital payments, which is a strong incentive to provide complete data, and allowed us to explore costs of subacromial decompression in England.
- We provide international comparisons of the use of subacromial decompression surgery.
- Our data are from 2007/08 onwards, so we under-estimate the amount spent on subacromial decompression prior to publication of major clinical trial results (CSAW and FIMPACT).
- There may be additional factors influencing surgery rates which we have not controlled for (e.g. private health insurance coverage).

For peer review only

INTRODUCTION

Health and social care services are 'straining at the seams' following increasing demand for services from an ageing population with more complex needs.[1] In England, over 200 Clinical Commissioning Groups (CCGs) have a budget to purchase health services for their local populations.[2] Hospital care currently accounts for 48.5% (£74 billion) of government health expenditure in the UK.[3] It is vital that commissioners make evidence-based decisions to maximise the effectiveness of this hospital care budget to benefit the overall health of the population.

Medicines must be licensed for use for a particular condition, requiring pharmaceutical companies to provide evidence of effectiveness from clinical trials to relevant agencies such as the Medicines and Healthcare products Regulatory Agency (MHRA) in the UK,[4] European Medicines Agency (EMA) in the EU, or the Food and Drug Administration (FDA) in the United States.[5] In the UK, the National Institute for Health and Care Excellence (NICE) also evaluates the cost-effectiveness of many medicines and does not recommend those which do not provide value. These regulatory processes have their limitations,[6] but require robust evidence for the introduction of new treatments. The quality of evidence required to introduce new surgical procedures is not as strict as for medicines,[4 5] in part because no specific product such as a drug or device is involved; it can be difficult to categorise procedures as 'new' rather than modifications; and outcomes may depend on the skill of the practitioner as well as the procedure itself.[4] Once introduced, use of procedures can spread by clinical consensus,[5] and established practice and clinical evidence often take many years to be updated.[7 8]

NHS England has recently commissioned a consultation regarding the use of 17 hospital procedures,[9] one of which is subacromial decompression for shoulder pain. Shoulder pain is common, with a lifetime prevalence of up to 66.7% [10]. Most of these cases (up to 70%) are related to rotator cuff tears or subacromial pain.[11] Subacromial pain is often considered to be caused by bony 'spurs' forming on the acromion, part of the shoulder blade, leading to inflammation in the surrounding

For peer review only - http://bmjopen.bmj.com/site/about/guidelines.xhtml

bursa and tendons.[12 13] Subacromial decompression removes the bony spur on the acromion and releases the coracohumeral ligament.[13 14] There has been a rapidly increasing use of subacromial decompression in England, with over 21,000 procedures carried out in 2009/10.[13]

Two recent multi-centre randomised controlled trials (RCTs) have questioned the effectiveness of subacromial decompression for shoulder pain.[15 16] The CSAW trial,[12 15] recruiting in England from 2012 to 2015, compared arthroscopic subacromial decompression surgery, placebo (investigational shoulder arthroscopy), and no treatment.[15] It found no difference in shoulder function after six months between the arthroscopic subacromial decompression group and the arthroscopy only (placebo) group, with a small, non-clinically significant benefit of surgery over the no treatment control. The FIMPACT trial,[16] recruiting in Finland from 2005 to 2013, compared subacromial decompression with placebo surgery and exercise therapy and echoed the results of CSAW, extending them to two years follow-up. The CSAW and FIMPACT trials seriously question whether the resources invested in subacromial decompression represent good value for money for the NHS. As a result, a recent BMJ article made a strong recommendation against subacromial decompression surgery for chronic shoulder pain.[17]

In this study we use subacromial decompression for shoulder pain as an example to explore the relationship between evolving evidence and clinical practice for hospital procedures, including how many procedures were performed over the last 10 years and how much money was spent before RCT evidence raised questions about the procedure's value; how procedure rates compare to other countries; and how the NHS might reduce the numbers of these procedures.

For peer review only - http://bmjopen.bmj.com/site/about/guidelines.xhtml

METHODS

Data sources

Subacromial decompression procedures were identified using the 'admitted patient care' hospital episode statistics (HES-APC). HES is a routinely collected dataset that records all episodes of care provided to patients admitted (day case or inpatient) to NHS hospitals in England and NHS-funded patients treated in the independent sector.[18 19] Each episode in HES represents a period of care under one consultant team. Up to 20 diagnoses are recorded per episode using the International Classification of Diseases (ICD) version 10. Up to 24 clinical procedures per episode may be recorded using Office of Population, Censuses and Surveys (OPCS) (fourth revision) codes. HES also includes the Lower Super Output Area (LSOA) of residence for each patient.[20]

Identifying subacromial decompression

We extracted anonymised, individual episodes in the HES-APC (2007/8 to 2016/17) dataset. We used diagnosis and procedure codes[13] (Figure 1) to identify subacromial decompression. A small number of patients received multiple shoulder procedure episodes on the same day (0.3% of all episodes). When these were for the same procedure with the same laterality (0.25% of all episodes), we assumed coding error duplication so excluded the episodes. If a procedure was marked as bilateral (0.6%), this was counted as two procedures. We excluded patients who were not resident in England.

Estimating procedure rates

National trends over time were estimated using directly standardised procedure rates [21] (per 100,000 population), with the population of England in 2016 as our standard population. For comparison of smaller areas, we estimated indirectly standardised rates[22] per 100,000 population,

For peer review only - http://bmjopen.bmj.com/site/about/guidelines.xhtml

using the same standard population, and adjusting for deprivation and ethnicity (see Appendix A for more details).

Estimating procedure costs

Costs were estimated for each financial year by linking Healthcare Resource Group (HRG) codes for each admission in HES with the Department of Health Payment by Results National Tariffs for the appropriate financial year;[23-32] see Appendix A for more details.

International comparisons

A search of Medline and the Cumulative Index of Nursing and Allied Health (CINAHL) databases was conducted for the terms “acromioplasty” or “subacromial decompression” in conjunction with “incidence” or “prevalence” or “epidemiology”. One author (TJ) screened the results for articles including rates of subacromial decompression contemporary with our data, and further screened cited articles within included studies, as well as articles which cited included studies.

All statistical analyses were conducted using Stata/MP 14.2 for Windows and we mapped variation in procedure rates across England in 2016/17 using ArcGIS ArcMap 10.5.1 for Desktop.

Patient and public involvement

Patients involved in the CSAW trial reviewed this manuscript; they were interested by the results and the cost-focussed perspective.

For peer review only

RESULTS

The use of subacromial decompression in England

There were 15,112 subacromial decompression procedures (30 per 100,000 population) in 2007/8, rising to 28,802 procedures (52 per 100,000 population) in 2016/17 (Figure 2), excluding those done in combination with rotator cuff repair. This represents a 91% increase in the number of subacromial decompressions over 10 years, with 266,692 procedures carried out in total. Most of this increase took place before 2011/12, and procedure rates have slightly decreased between 2011/12 and 2016/17. The use of subacromial decompression in combination with rotator cuff repair has continued to increase since the early 2000s, more notably from 2006/7 onwards. Whilst the gender balance and age of those having shoulder surgery have remained steady over the last decade, the proportion of procedures conducted as day cases, using arthroscopy, and/or by independent (i.e. non-NHS) providers, have all increased (Table 1).

Table 1: Descriptive information for subacromial decompression patients, 2007/08 and 2016/17

	2007/08	*2016/17*
Procedure Count	15,112	28,802
%women	51.0	52.0
Age in years (SD)	54.94 (12.55)	54.89 (12.39)
% Arthroscopy	39.0	94.1
% Independent Providers	2.4	31.9
% Day-case	51.0	79.3

The cost of subacromial decompression in England

In 2016/17, the median cost of an elective admission for subacromial decompression alone was £4,476. The cost of subacromial decompression in England rose from £33 million in 2007/08 to £125 million in 2016/17. Over the 10-year period between 2007/8 and 2016/17 just under £1.1 billion was spent on subacromial decompression (excluding procedures done in combination with rotator cuff repair).

For peer review only

Variation in use of subacromial decompression in England

In 2016/17 there was substantial variation in procedure rates between CCGs, after adjusting for age, sex, deprivation, and ethnicity profiles (Figure 3). The map demonstrates pockets of very high use (>150% of the expected rate), for example in the Reading area, Wiltshire, and East Lincolnshire. There were also areas where procedure rates were less than 50% of the expected rate, such as in Worcestershire, Gloucestershire, Swindon, and North Norfolk. In 2016/17 the ratio of procedure rates between a 'high use' CCG at the 90th percentile and a 'low use' CCG at the 10th percentile was 2.7 (95% CI: 2.2-3.4). This ratio has decreased since 2007/8, when the ratio was 3.6 (95% CI: 2.2-6.1); see Table 2.

Table 2: 90/10 percentile ratios for directly age-sex standardised rates of subacromial decompression by CCG, England, 2007/8-2016/17

Year	90th Pct	10th Pct	90/10 Ratio (95% CI*)
2007/08	53	15	3.6 (2.2-6.1)
2008/09	55	16	3.3 (2.1-5.2)
2009/10	72	27	2.6 (2.0-3.5)
2010/11	87	33	2.6 (1.9-3.6)
2011/12	89	36	2.5 (2.0-3.1)
2012/13	90	33	2.7 (2.0-3.7)
2013/14	88	34	2.6 (2.1-3.3)
2014/15	89	33	2.7 (2.0-3.7)
2015/16	81	33	2.5 (1.4-4.3)
2016/17	83	30	2.7 (2.2-3.4)

**Confidence intervals for rate ratios*[33]

International comparison of rates of subacromial decompression

Table 3 shows rates of subacromial decompression in the most recent year available from England, Finland, Florida State, New York State, and Western Australia. Rates in England were lower, often only half, that of other countries. For subacromial decompression alone, the procedure rates were lower in England (52 per 100,000 in 2016/17) than Western Australia (roughly 115 per 100,000 in 2013),[34] Florida State (130 per 100,000 in 2007),[35] and Finland (131 per 100,000 in 2011).[36]

For peer review only

For subacromial decompression combined with rotator cuff repair, rates were lower in England (80 per 100,000 in 2016/17) than in New York State a decade earlier (102 per 100,000 in 2006).[37]

Figure 2 compares trends in rates of subacromial decompression in England, Finland, Florida State, New York State, and Western Australia. The rate of increase for subacromial decompression observed in our study (x2 between 2007/8 and 2016/17) was similar to Western Australia (x2 between 2001 and 2013),[34] Finland (x2.2 between 1998 and 2007),[36] and New York State (x2.5 between 1996 and 2006),[37] but lower than Florida State (x4.4 between 2003 and 2007).[35] The use of subacromial decompression in Finland peaked in 2007 and has since been declining, at least in publicly-funded hospitals, which has been attributed to accumulating evidence that it is no more clinically effective than non-surgical alternatives.[36]

Table 3: International comparisons of age-sex-standardised rates of subacromial decompression

**Article**	**Country**	**Data Year**	**SAD Rate (per 100,000 population)**
Thorpe et al. (2016)	Western Australia	2013	~115
Paloneva et al. (2015)	Finland	2011	131
Vitale et al. (2010)	New York State	2006	102
Iyengar et al. (2014)	Florida State	2007	~130
Our Data (inc. RCR)	England	2016/17	80
Our Data (exc. RCR)	England	2016/17	52

Notes: Numbers for Thorpe et al (2016) and Iyengar et al. (2014) were estimated from a graph; New York State data is for subacromial decompression with/without rotator cuff repair; SAD = Subacromial Decompression; RCR = Rotator Cuff Repair[34-37]

For peer review only

DISCUSSION

Statement of principal findings

NHS England carries out nearly 30,000 subacromial decompression operations per year, at an annual cost of over £125 million. Between 2007/08 and 2016/17, 266,692 subacromial decompression procedures were carried out in England costing nearly £1.1 billion, before the publication of CSAW and FIMPACT trial results prompted questions about the clinical benefit of the procedure. Rates of subacromial decompression alone in England have gradually declined since 2011/12, although an increasing number are carried out in combination with rotator cuff repair. There was large variation between CCGs in England, even after adjustment for demographic variables, with 'high-use' areas carrying out nearly three times as many procedures as 'low-use' areas. Procedure rates in England were notably lower than other countries, arguing against any levelling of procedure rates being due to saturation of 'demand' for shoulder surgery.

Strengths and weaknesses of the study

Our study used a national, longitudinal dataset over a 10-year period covering all NHS secondary care providers in England. Hospital Episode Statistics are administrative rather than specifically designed for research. However, HES is also linked to payments for hospitals, which is a strong incentive to provide complete data, and allowed us to produce what we believe is the first exploration of costs associated with subacromial decompression in England. Payment by results tariffs are based on average national costs and may not reflect precise costs for each hospital admission. Population denominators, and linkage to the indices of multiple deprivation and census ethnicity data, allowed us to investigate trends and variations in procedure rates standardised on age, sex, deprivation and ethnicity. HES data records patients' area of residence, so we compared procedure rates based on place of residence rather than place of treatment. There may be other factors influencing rates which we have not controlled for (e.g. private health insurance coverage).

For peer review only - http://bmjopen.bmj.com/site/about/guidelines.xhtml

HES does not record procedures which are privately funded and provided, meaning our surgery rates are an under-estimate of the population rate. We only provide cost data from 2007/08 onwards, so we under-estimate the amount spent on subacromial decompression prior to publication of the CSAW and FIMPACT trial results. International estimations of procedure rates do not use identical definitions of procedures and inclusion/exclusion criteria, but should be broadly comparable.

Implications for policymakers and clinicians

NHS England spent over £1 billion on subacromial decompression during the last 10 years without having compelling evidence of clinical effectiveness or cost-effectiveness. Rates of subacromial decompression were already rising rapidly from 2000/01 onwards.[13] It seems plausible that increasing awareness of concerns about the effectiveness of subacromial decompression surgery and well-known recruitment to the CSAW trial tempered the rise in use of this surgery in England, otherwise more may have been spent. The CSAW trial involved 51 surgeons in 30 centres throughout the UK and was widely advertised and discussed amongst shoulder surgeons and shoulder physiotherapists. Extensive consultation was carried out by the trial team prior to and during the trial, including presentations at national meetings surveys and visits to individual surgeons and centres.[38] A similar plateau/decrease in procedures was observed in Finland after the commencement of the FIMPACT study in 2005 which involved only 3 centres in Finland (Figure 2). However, it took well over a decade of increasing subacromial decompression use for clinical trial groups to randomise a few hundred patients (313 patients for CSAW [15] and 210 in FIMPACT[16]) to investigate its effectiveness. This delay may be due to perceived difficulties in recruiting patients to surgical trials with non-surgical comparators (e.g. UKUFF[39]), as well as known challenges of conducting surgical RCTs.[40] Methods to optimise recruitment, as used in CSAW and other trials,[41] are available to support the completion of such 'difficult' trials;[42] this should not now be a barrier to rapidly initiating trials to provide robust evidence about surgical interventions before they become widespread. More time is needed to see the longer-term impact of publication of the CSAW and

For peer review only - http://bmjopen.bmj.com/site/about/guidelines.xhtml

FIMPACT results on subacromial decompression rates, both in the UK and internationally. It is also worth noting the increasing tendency in England to carry out subacromial decompression in combination with rotator cuff repair, and any impact on this following dissemination of the CSAW/FIMPACT results.

The National Institute for Health and Care Excellence (NICE) requires evidence of cost-effectiveness to recommend new medicines to be paid for by the NHS. It is unclear why the bar for introducing expensive surgical procedures should be significantly lower. A balance needs to be struck between supporting innovation in surgical procedures and preventing unnecessary treatment. New initiatives such as IDEAL (Idea, Development, Exploration, Assessment, Long-term Follow-up, Improving the Quality of Research in Surgery)[43] aim to provide such a regulatory framework for introducing new interventions.

It is important that new evidence is disseminated quickly without causing inequities in access to care. NICE published an updated Clinical Knowledge Summary for shoulder pain in April 2017[44] incorporating information from a commissioning guide published by the Royal College of Surgeons.[45] This recommended a range of conservative treatments from physiotherapy to corticosteroid injections, before surgery. However, many CCGs introduced their own criteria-based policies for access to shoulder surgery (e.g. through Individual Funding Requests)[46] at different times and with different details, underlining the extent to which insufficient evidence may drive clinical and commissioner uncertainty,[47] and possibly leading to the wide variations shown across CCGs in our data. Where scientific evidence is applicable nationally or internationally, it would seem more efficient and appropriate to apply national policies to inform optimal use and encourage further research. There is a need to improve techniques for empirically-informed policy development in collaboration with relevant stakeholders.[48 49] It is also important to note that certain patients may still benefit from surgery. Further well conducted research is needed to understand if specific sub-groups of patients might benefit from subacromial decompression surgery.

For peer review only

For peer review only - http://bmjopen.bmj.com/site/about/guidelines.xhtml

Despite the criticisms provided above, England has lower rates of shoulder surgery than other countries. The reasons for this are uncertain but could be due to differences in the health systems (e.g. GP gatekeeping of services), access to surgery and hospital reimbursement. Additionally, the National Institute of Health Research in England has funded major clinical trials on shoulder surgery,[15 39] as well as other procedures;[50 51] and is about to fund a further clinical trial to compare surgery with placebo surgery for partial thickness rotator cuff tears.[52] Whilst the UK's national regulatory processes are imperfect, they may provide examples to learn from. However, these processes did not sufficiently constrain the use of subacromial decompression, a procedure later found to have little clinical benefit.

There have been several other controversies regarding the lack of effectiveness of procedures which have become commonplace. One example is the use of stents to open narrowed arteries for treatment of stable angina (chest pain). Around half a million people receive stents for stable angina each year in the US and Europe,[53] but a recent (RCT) including a placebo intervention found no difference in chest pain outcomes between inserting a stent and using standard medications.[54] Another example is arthroscopy to clean out the knee joint, on which around $4 billion is spent each year in the US.[55] Recent RCTs,[56 57] including one using a placebo procedure as a comparison,[57] found no evidence of effectiveness to justify the spending. Whilst we use subacromial decompression as an example in this study, our observations are likely to apply to interventional procedures more generally.

Unanswered questions and future research

The example of subacromial decompression highlights that, in the absence of rigorous evaluation, costly interventions can proliferate over a long period of time. To maximise limited resources, it is vital that methods are developed to identify promising procedures early and commission trials to examine their value, as well as identify existing health technologies that may be ineffective, leading to over-treatment and wasting of resources.

For peer review only - http://bmjopen.bmj.com/site/about/guidelines.xhtml

There is an opportunity for a natural experiment exploring the impact of the results of the CSAW and FIMPACT trials[15 16] on the development of CCG policies, national guidelines, and clinical decision-making with surgeons and patients. It is arguable that we should now see swift reductions in the use of subacromial decompression; research studies could help enhance the transfer of knowledge from trials into clinical practice.

Conclusions

NHS England pays for nearly 30,000 shoulder subacromial decompression procedures each year at an annual cost of over £125 million, with little evidence that they are effective or cost-effective. The rates of this operation in other countries are even higher. This raises serious questions around the regulatory and professional processes governing the adoption and widespread use of surgical interventions. High quality RCTs should be funded early to examine the effectiveness and cost-effectiveness of expensive procedures using methods to optimise recruitment, and robust processes should be developed to reduce the use of ineffective procedures.

For peer review only - http://bmjopen.bmj.com/site/about/guidelines.xhtml

For peer review only

Author Statement

This publication is the work of the authors, who serve as guarantors for the contents of this paper. TJ contributed to study design, data cleaning, data analysis, interpretation of results and writing the manuscript. MJL contributed to study design, data cleaning, interpretation of results and writing the manuscript. AC contributed to study design, interpretation of results and writing the manuscript. DB, LR, and JD contributed to interpretation of results and writing the manuscript. WH contributed to study conceptualization, study design, interpretation of results and writing the manuscript. TJ had full access to the data in the study and takes responsibility for the integrity of the data and the accuracy of the data analysis. The authors would like to thank two patients from the CSAW trial, Carol Brennan and Dair Farrer-Hockley, who took the time to read and comment on this manuscript.

Funding

This research was funded by the National Institute for Health Research (NIHR) Collaboration for Leadership in Applied Health Research and Care West (NIHR CLAHRC West). The views expressed in this article are those of the author(s) and not necessarily those of the NHS, the NIHR, or the Department of Health and Social Care.

Ethnical Approval

We were provided with routinely-collected Hospital Episode Statistics data under licence from NHS Digital (DARS-NIC-17875-X7K1V). The licence allows us to use the information under Section 261 of the Health and Social Care Act 2012, 2(b)(ii): "after taking into account the public interest as well as the interests of the relevant person, considers that it is appropriate for the information to be disseminated".

Data Sharing

This study is based in part on data from the Hospital Episode Statistics (HES) obtained under licence (DARS-NIC-17875-X7K1V) from NHS Digital (previously the Health and Social Care Information

For peer review only

For peer review only - http://bmjopen.bmj.com/site/about/guidelines.xhtml

Centre); *Copyright © 2018, re-used with the permission of The Health & Social Care Information Centre. All rights reserved*. The data are provided by patients and collected by the NHS as part of their care and support. HES data can be accessed via NHS Digital: https://digital.nhs.uk/services/data-access-request-service-dars

Transparency

The manuscript's guarantor (TJ) affirms that the manuscript is an honest, accurate, and transparent account of the study being reported; that no important aspects of the study have been omitted; and that any discrepancies from the study as originally planned have been explained.

Competing Interests

All authors have completed the ICMJE uniform disclosure form at www.icmje.org/ coi_disclosure.pdf and declare: TJ and JD had financial support from NIHR CLAHRC West for the submitted work; no financial relationships with any organisations that might have an interest in the submitted work in the previous three years; no other relationships or activities that could appear to have influenced the submitted work.

For peer review only

REFERENCES

1. Care Quality Commission. The state of health care and adult social care in England, 2017. Available from: http://www.cqc.org.uk/sites/default/files/20171017_stateofcare1617_report.pdf.
2. NHS Clinical Commissioners. About CCGs 2017 [Available from: https://www.nhscc.org/ccgs/ accessed 3rd November 2017.
3. Office for National Statistics. UK Health Accounts: 2016, 2018. Available from: https://www.ons.gov.uk/peoplepopulationandcommunity/healthandsocialcare/healthcaresystem/bulletins/ukhealthaccounts/2016.
4. Campbell B. Regulation and safe adoption of new medical devices and procedures. *Brit Med Bull* 2013;107(1):5-18. doi: 10.1093/bmb/ldt022
5. Darrow JJ. Explaining the absence of surgical procedure regulation. *Cornell Journal of Law and Public Policy* 2017;27(1):189-206.
6. Hollingworth W, Jones T, Reeves BC, et al. A longitudinal study to assess the frequency and cost of antivascular endothelial therapy, and inequalities in access, in England between 2005 and 2015. *Bmj Open* 2017;7(10) doi: 10.1136/bmjopen-2017-018289
7. Tatsioni A, Bonitsis NG, Ioannidis JPA. Persistence of contradicted claims in the literature. *Jama-J Am Med Assoc* 2007;298(21):2517-26. doi: DOI 10.1001/jama.298.21.2517
8. Jevne J. The sexy scalpel: unnecessary shoulder surgery on the rise. *Brit J Sport Med* 2015;49(16):1031-32. doi: 10.1136/bjsports-2015-094589
9. NHS England, NHS Clinical Commissioners, Academy of Medical Royal Colleges, et al. Evidence-Based Interventions: Consultation Document: NHS England, 2018.
10. Luime JJ, Koes BW, Hendriksen IJM, et al. Prevalence and incidence of shoulder pain in the general population; a systematic review. *Scand J Rheumatol* 2004;33(2):73-81. doi: 10.1080/03009740310004667
11. Mitchell C, Adebajo A, Hay E, et al. Shoulder pain: diagnosis and management in primary care. *Brit Med J* 2005;331(7525):1124-28a. doi: DOI 10.1136/bmj.331.7525.1124
12. Beard D, Rees J, Rombach I, et al. The CSAW Study (Can Shoulder Arthroscopy Work?) - a placebo-controlled surgical intervention trial assessing the clinical and cost effectiveness of arthroscopic subacromial decompression for shoulder pain: study protocol for a randomised controlled trial. *Trials* 2015;16 doi: 10.1186/s13063-015-0725-y
13. Judge A, Murphy RJ, Maxwell R, et al. Temporal trends and geographical variation in the use of subacromial decompression and rotator cuff repair of the shoulder in England. *Bone Joint J* 2014;96b(1):70-74. doi: 10.1302/0301-620x.96b1.32556
14. Chalmers PN, Romeo AA. Arthroscopic Subacromial Decompression and Acromioplasty. *Jbjs Essent Surg Tec* 2016;6(2) doi: 10.2106/JBJS.ST.O.00011
15. Beard DJ, Rees JL, Cook JA, et al. Arthroscopic subacromial decompression for subacromial shoulder pain (CSAW): a multicentre, pragmatic, parallel group, placebo-controlled, three-group, randomised surgical trial. *The Lancet* 2017 doi: 10.1016/s0140-6736(17)32457-1
16. Paavola M, Malmivaara A, Taimela S, et al. Subacromial decompression versus diagnostic arthroscopy for shoulder impingement: randomised, placebo surgery controlled clinical trial. *Bmj-Brit Med J* 2018;362 doi: 10.1136/bmj.k2860
17. Vandvik PO, Lähdeoja T, Ardern C, et al. Subacromial decompression surgery for adults with shoulder pain: a clinical practice guideline. *BMJ* 2019;364:l294. doi: 10.1136/bmj.l294
18. Health and Social Care Information Centre. Hospital Episode Statistics (HES) Analysis Guide, 2015.
19. Herbert A, Wijlaars L, Zylbersztejn A, et al. Data Resource Profile: Hospital Episode Statistics Admitted Patient Care (HES APC). *Int J Epidemiol* 2017;46(4):1093-+. doi: 10.1093/ije/dyx015
20. NHS Digital. Lower Layer Super Output Area . Available from: https://www.datadictionary.nhs.uk/data_dictionary/nhs_business_definitions/l/lower_layer_super_output_area_de.asp?shownav=1 accessed 14 May 2018.

For peer review only - http://bmjopen.bmj.com/site/about/guidelines.xhtml

21. Standardization: a classic epidemiological method for the comparison of rates. *Epidemiol Bull* 2002;23(3):9-12. [published Online First: 2003/03/01]

22. Anon. Standardization: a classic epidemiological method for the comparison of rates. *Epidemiological Bulletin* 2002;23(3):9-12.

23. Department of Health. Payment by Results National Tariffs 2016/17, 2016. Available from: https://www.gov.uk/government/publications/nhs-national-tariff-payment-system-201617 accessed 30 November 2017.

24. Department of Health. Payment by Results Enhanced Tariff Option 2015/16, 2015. Available from: http://webarchive.nationalarchives.gov.uk/20161103233943/https://www.england.nhs.uk/resources/pay-syst/tariff-guide/ accessed 30 November 2017.

25. Department of Health. Payment by Results National Tariffs 2014/15, 2014. Available from: https://www.gov.uk/government/publications/national-tariff-payment-system-2014-to-2015 accessed 30 November 2017.

26. Department of Health. Payment by Results National Tariffs 2013/14, 2013. Available from: https://www.gov.uk/government/publications/payment-by-results-pbr-operational-guidance-and-tariffs accessed 30 November 2017.

27. Department of Health. Payment by Results National Tariffs 2012/13, 2012. Available from: https://www.gov.uk/government/publications/confirmation-of-payment-by-results-pbr-arrangements-for-2012-13 accessed 30 November 2017.

28. Department of Health. Payment by Results National Tariffs 2011/12, 2011. Available from: http://webarchive.nationalarchives.gov.uk/20130507170152/https://www.gov.uk/government/publications/confirmation-of-payment-by-results-pbr-arrangements- for-2011-12 accessed 30 November 2017.

29. Department of Health. Payment by Results National Tariffs 2010/11, 2010. Available from: https://data.gov.uk/dataset/payment-by-results-2010-11-national-tariff-information accessed 30 November 2017.

30. Department of Health. Payment by Results National Tariffs 2009/10, 2009. Available from: http://webarchive.nationalarchives.gov.uk/+/http://www.dh.gov.uk/en/Publicationsandstatistics/Publications/PublicationsPolicyAndGuidance/DH_094091 accessed 30 November 2017.

31. Department of Health. Payment by Results National Tariffs 2008/09, 2008. Available from: http://webarchive.nationalarchives.gov.uk/+/http://www.dh.gov.uk/en/Managingyourorganisation/Financeandplanning/NHSFinancialReforms/DH_081238 accessed 30 November 2017.

32. Department of Health. Payment by Results National Tariffs 2007/08, 2007. Available from: http://webarchive.nationalarchives.gov.uk/+/http://www.dh.gov.uk/en/Publicationsandstatistics/Publications/PublicationsPolicyAndGuidance/DH_062914 accessed 30 November 2017.

33. Kirkwood BR, Sterne JA. Rate Ratios. Essential Medical Statistics. Oxford, UK: Blackwell Science Ltd 2003:241-42.

34. Thorpe A, Hurworth M, O'Sullivan P, et al. Rising trends in surgery for rotator cuff disease in Western Australia. *Anz J Surg* 2016;86(10):801-04. doi: 10.1111/ans.13691

35. Iyengar JJ, Samagh SP, Schairer W, et al. Current Trends in Rotator Cuff Repair: Surgical Technique, Setting, and Cost. *Arthroscopy* 2014;30(3):284-88. doi: 10.1016/j.arthro.2013.11.018

36. Paloneva J, Lepola V, Karppinen J, et al. Declining incidence of acromioplasty in Finland. *Acta Orthop* 2015;86(2):220-24. doi: 10.3109/17453674.2014.977703

37. Vitale MA, Arons RR, Hurwitz S, et al. The Rising Incidence of Acromioplasty. *J Bone Joint Surg Am* 2010;92a(9):1842-50. doi: 10.2106/Jbjs.I.01003

For peer review only - http://bmjopen.bmj.com/site/about/guidelines.xhtml

38. Wartolowska K, Beard DJ, Carr AJ. Attitudes and Beliefs about Placebo Surgery among Orthopedic Shoulder Surgeons in the United Kingdom. *Plos One* 2014;9(3) doi: 10.1371/journal.pone.0091699
39. Carr A, Cooper C, Campbell MK, et al. Effectiveness of open and arthroscopic rotator cuff repair (UKUFF) A RANDOMISED CONTROLLED TRIAL. *Bone Joint J* 2017;99b(1):107-15. doi: 10.1302/0301-620x.99b1.Bjj-2016-0424.R1
40. Cook JA, Ramsay CR, Norrie J. Recruitment to publicly funded trials - Are surgical trials really different? *Contemp Clin Trials* 2008;29(5):631-34. doi: 10.1016/j.cct.2008.02.005
41. Rooshenas L, Scott LJ, Blazeby JM, et al. The Quintet Recruitment Intervention supported five randomized trials to recruit to target: a mixed-methods evaluation, 2018. Available from: https://doi.org/10.1016/j.jclinepi.2018.10.004.
42. Donovan JL, Rooshenas L, Jepson M, et al. Optimising recruitment and informed consent in randomised controlled trials: the development and implementation of the Quintet Recruitment Intervention (QRI). *Trials* 2016;17 doi: 10.1186/s13063-016-1391-4
43. Hirst A, Philippou Y, Blazeby J, et al. No Surgical Innovation Without Evaluation: Evolution and Further Development of the IDEAL Framework and Recommendations. *Annals of Surgery* 2018 doi: 10.1097/SLA.0000000000002794
44. National Institute for Health and Care Excellence. Shoulder Pain, 2017.
45. The Royal College of Surgeons of England. Commissioning guide: Subacromial shoulder pain, 2014.
46. Iacobucci G. Pressure on NHS finances drives new wave of postcode rationing. *Bmj-Brit Med J* 2017;358 doi: 10.1136/bmj.j3190
47. Wennberg JE, Barnes BA, Zubkoff M. Professional Uncertainty and the Problem of Supplier-Induced Demand. *Soc Sci Med* 1982;16(7):811-24. doi: Doi 10.1016/0277-9536(82)90234-9
48. Hollingworth W, Rooshenas L, Busby J, et al. Using clinical practice variations as a method for commissioners and clinicians to identify and prioritise opportunities for disinvestment in health care: a cross-sectional study, systematic reviews and qualitative study. *Health Services and Delivery Research* 2015 doi: 10.3310/hsdr03130
49. Rooshenas L, Owen-Smith A, Hollingworth W, et al. "I won't call it rationing ...": An ethnographic study of healthcare disinvestment in theory and practice. *Soc Sci Med* 2015;128:273-81. doi: 10.1016/j.socscimed.2015.01.020
50. Hamdy FC, Donovan JL, Lane JA, et al. 10-Year Outcomes after Monitoring, Surgery, or Radiotherapy for Localized Prostate Cancer. *New Engl J Med* 2016;375(15):1415-24. doi: 10.1056/NEJMoa1606220
51. Rubie I, Haighton C, O'Hara J, et al. The NAtional randomised controlled Trial of Tonsillectomy IN Adults (NATTINA): a clinical and cost-effectiveness study: study protocol for a randomised control trial. *Trials* 2015;16 doi: 10.1186/s13063-015-0768-0
52. National Institute for Health Research. 18/109 Management of Partial Thickness Rotator-Cuff Tears, 2018. Available from: https://www.nihr.ac.uk/funding-and-support/funding-opportunities/18109-management-of-partial-thickness-rotator-cuff-tears/89052018.
53. Belluz J. Thousands of heart patients get stents that may do more harm than good: Vox; 2017. Available from: https://www.vox.com/science-and-health/2017/11/3/16599072/stent-chest-pain-treatment-angina-not-effective.
54. Al-Lamee R, Thompson D, Dehbi HM, et al. Percutaneous coronary intervention in stable angina (ORBITA): a double-blind, randomised controlled trial. *Lancet* 2018;391(10115):31-40. doi: 10.1016/S0140-6736(17)32714-9
55. Epstein D. When evidence says no, but doctors say yes: ProPublica; 2017. Available from: https://web.archive.org/web/20170316151510/https://www.propublica.org/article/when-evidence-says-no-but-doctors-say-yes2018.
56. Katz JN, Brophy RH, Chaisson CE, et al. Surgery versus Physical Therapy for a Meniscal Tear and Osteoarthritis. *New Engl J Med* 2013;368(18):1675-84. doi: 10.1056/NEJMoa1301408

For peer review only

For peer review only - http://bmjopen.bmj.com/site/about/guidelines.xhtml

559 57. Sihvonen R, Paavola M, Malmivaara A, et al. Arthroscopic Partial Meniscectomy versus Sham Surgery for a Degenerative Meniscal Tear. *New Engl J Med* 2013;369(26):2515-24. doi: 10.1056/NEJMoa1305189

For peer review only

For peer review only - http://bmjopen.bmj.com/site/about/guidelines.xhtml

FIGURE CAPTIONS

Figure 1. ICD-10 and OPCS-4 codes used to define subacromial decompression[13]

Figure 2. Directly standardised rates (per 100,000 people) of subacromial decompression in England, Finland, New York State USA, Florida State USA, and Western Australia

Notes for Figure 2. England data prior to 2007 is taken from Judge et al.[13]; New York State data is for subacromial decompression with or without rotator cuff repair, whilst data for Florida State, Finland and Western Australia is for subacromial decompression alone [34-37]; RCR = Rotator Cuff Repair

Figure 3. Indirectly standardised rates of subacromial decompression by CCG in England, 2016/17

For peer review only

For peer review only - http://bmjopen.bmj.com/site/about/guidelines.xhtml

Any of these ***diagnosis*** *codes in any position*		*Any of these* ***procedure*** *codes in any position*
M75.1 Rotator cuff syndrome	In combination with...	W84.8 Other specified therapeutic endoscopic operations on other joint structure
M75.3 Calcific tendinitis of shoulder		Y52.8 Other specified approach to organ through other opening
M75.4 Impingement syndrome of shoulder		Y76.7 Arthroscopic approach to joint
M75.5 Bursitis of shoulder		W84.4 Endoscopic decompression of joint

OR

This ***procedure*** *code in any position*
O29.1 Subacromial decompression

Figure 1. ICD-10 and OPCS-4 codes used to define subacromial decompression[13]

102x65mm (300 x 300 DPI)

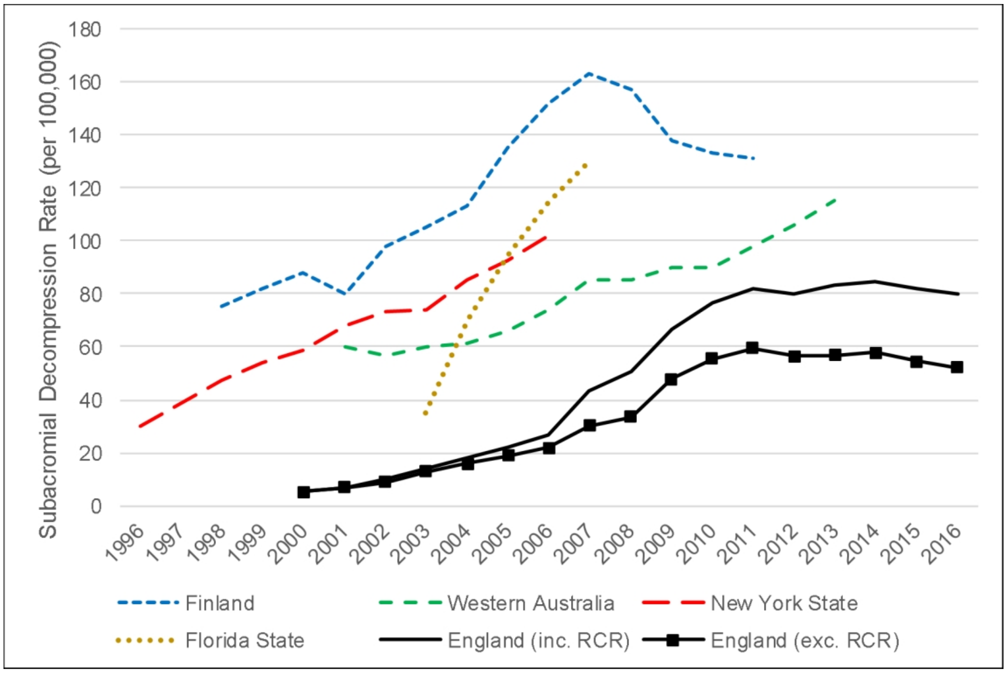

Figure 2. Directly standardised rates (per 100,000 people) of subacromial decompression in England, Finland, New York State USA, Florida State USA, and Western Australia

Notes for Figure 2. England data prior to 2007 is taken from Judge et al.[13]; New York State data is for subacromial decompression with or without rotator cuff repair, whilst data for Florida State, Finland and Western Australia is for subacromial decompression alone [34-37]; RCR = Rotator Cuff Repair

150x99mm (300 x 300 DPI)

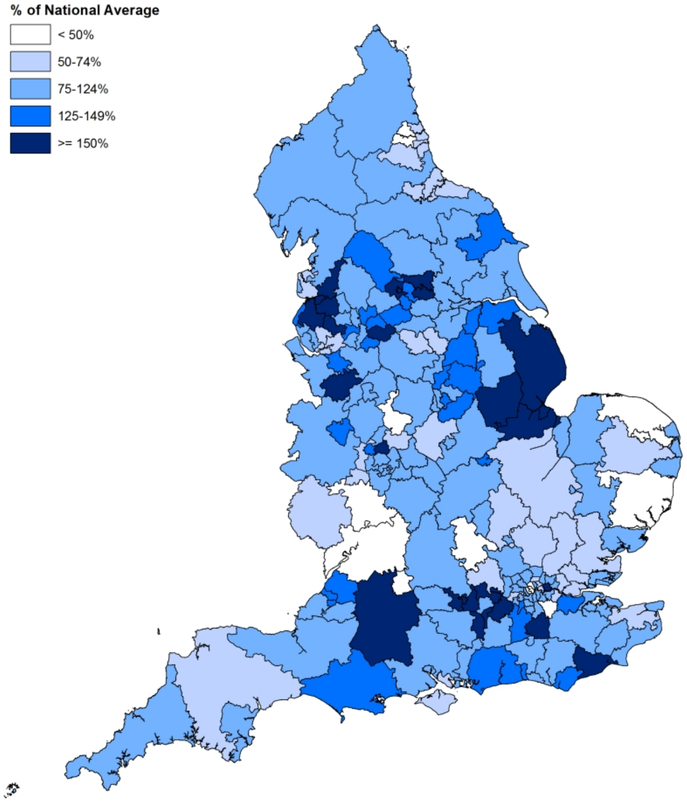

Figure 3. Indirectly standardised rates of subacromial decompression by CCG in England, 2016/17

210x296mm (300 x 300 DPI)

APPENDIX A: METHODOLOGICAL DETAILS

Estimating Procedure Rates

National trends over time were estimated using directly standardised procedure rates(1) (per 100,000 population), with the population of England in 2016 as our standard population. We first summed the number of shoulder procedures, grouped by sex, quintiles of age, and financial year. These procedure counts were used to calculate annual age-sex-specific rates, by dividing by the appropriate age-sex-specific mid-year populations of England(2) (e.g. for the 2012/13 financial year, the mid-2012 populations were used). We weighted the annual age-sex-specific rates according to the population distribution of England in 2016, to produce directly standardised rates for each year. The standardised rates for 2016/17 are the same as the crude rates.

For comparison of smaller areas, we estimated indirectly standardised rates(3) per 100,000 population. We first calculated age-sex-specific rates for England in 2016/17, then multiplied these rates by the age-sex-specific population for the area of interest(2, 4, 5) (e.g. CCG) and summed the results. This produced the expected number of patients and procedures for that area, if it were to have the same age-sex-specific rates as England. The expected number was then compared to the observed number of patients and procedures for that area. A Poisson regression model was fitted to the observed counts for each year, with the expected counts as an offset and socio-economic deprivation (using the overall score from the English Indices of Multiple Deprivation(6)) and ethnicity (% white British(7)) as predictive factors. The model was then used to predict new expected counts for each area based on deprivation and ethnicity, and form indirectly standardised procedure ratios (observed / expected).

For peer review only

Estimating Procedure Costs

Costs were estimated for each financial year by linking HRG codes for each admission in HES with the Department of Health Payment by Results National Tariffs for the appropriate financial year.(8-17) Enhanced Tariff Option (ETO) tariffs were applied for 2015/16 as, following a dispute, 88% of providers agreed to use ETO tariffs for that financial year.(18) The National Tariffs provide costs for day cases and longer stays, for both elective and non-elective admissions. They also provide additional daily costs for admissions that go above a threshold number of days (termed excess bed days), which varies for different types of admission. To calculate the cost of admission, we excluded admissions without a discharge date (used to calculate number of bed days) or without a HRG code that matched to the National Tariffs (0.7% excluded). We then applied the relevant national tariff or alternatively the best practice tariff where applicable (only for HRG code HB62C under specified circumstances) and added excess bed day costs (if there were any). Following this, the special service top-up for orthopaedic procedures was applied for each year.

References

1. Standardization: a classic epidemiological method for the comparison of rates. Epidemiol Bull. 2002;23(3):9-12.
2. Office for National Statistics. Lower Super Output Area Mid-Year Population Estimates. Available from: https://www.ons.gov.uk/peoplepopulationandcommunity/populationandmigration/populationestimates/datasets/lowersuperoutputareamidyearpopulationestimates.
3. Anon. Standardization: a classic epidemiological method for the comparison of rates. Epidemiological Bulletin. 2002;23(3):9-12.
4. Office for National Statistics. Lower Layer Super Output Area (2011) to Clinical Commissioning Group to Local Authority District (July 2015) Lookup in England: Office for National Statistics. Available from: http://ons.maps.arcgis.com/home/item.html?id=7de88cd2369d4006925de49e8f3f2e27.
5. Office for National Statistics. Output Area to Lower Layer Super Output Area to Middle Layer Super Output Area to Local Authority District (December 2011) Lookup in England and Wales. Available from: http://ons.maps.arcgis.com/home/item.html?id=baf6566ca08949c6bbd61ff81d9526da.
6. Government DoCaL. English Indices of Deprivation 2015. Available from: https://www.gov.uk/government/statistics/english-indices-of-deprivation-2015.

For peer review only

7. Nomis; Office for National Statistics. Ethnic group of the usual resident population of England and Wales as at census day, 27 March 2011. Available from: https://www.nomisweb.co.uk/census/2011/ks201ew.
8. Department of Health. Payment by Results National Tariffs 2016/17 2016. Available from: https://www.gov.uk/government/publications/nhs-national-tariff-payment-system-201617.
9. Department of Health. Payment by Results Enhanced Tariff Option 2015/16 2015. Available from: http://webarchive.nationalarchives.gov.uk/20161103233943/https://www.england.nhs.uk/resources/pay-syst/tariff-guide/.
10. Department of Health. Payment by Results National Tariffs 2014/15 2014. Available from: https://www.gov.uk/government/publications/national-tariff-payment-system-2014-to-2015.
11. Department of Health. Payment by Results National Tariffs 2013/14 2013. Available from: https://www.gov.uk/government/publications/payment-by-results-pbr-operational-guidance-and-tariffs.
12. Department of Health. Payment by Results National Tariffs 2012/13 2012. Available from: https://www.gov.uk/government/publications/confirmation-of-payment-by-results-pbr-arrangements-for-2012-13.
13. Department of Health. Payment by Results National Tariffs 2011/12 2011. Available from: http://webarchive.nationalarchives.gov.uk/20130507170152/https://www.gov.uk/government/publications/confirmation-of-payment-by-results-pbr-arrangements- for-2011-12.
14. Department of Health. Payment by Results National Tariffs 2010/11 2010. Available from: https://data.gov.uk/dataset/payment-by-results-2010-11-national-tariff-information.
15. Department of Health. Payment by Results National Tariffs 2009/10 2009. Available from: http://webarchive.nationalarchives.gov.uk/+/http://www.dh.gov.uk/en/Publicationsandstatistics/Publications/PublicationsPolicyAndGuidance/DH_094091.
16. Department of Health. Payment by Results National Tariffs 2008/09 2008. Available from: http://webarchive.nationalarchives.gov.uk/+/http://www.dh.gov.uk/en/Managingyourorganisation/Financeandplanning/NHSFinancialReforms/DH_081238.
17. Department of Health. Payment by Results National Tariffs 2007/08 2007. Available from: http://webarchive.nationalarchives.gov.uk/+/http://www.dh.gov.uk/en/Publicationsandstatistics/Publications/PublicationsPolicyAndGuidance/DH_062914.
18. Grasic K, Mason AR, Street A. Paying for the quantity and quality of hospital care: the foundations and evolution of payment policy in England. Health Econ Rev. 2015;5.

For peer review only

BMJ Open

A longitudinal study of use and cost of subacromial decompression surgery: the need for effective evaluation of surgical procedures to prevent overtreatment and wasted resources.

Journal:	*BMJ Open*
Manuscript ID	bmjopen-2019-030229.R1
Article Type:	Research
Date Submitted by the Author:	01-Jul-2019
Complete List of Authors:	Jones, Tim; University of Bristol, NIHR CLAHRC West<br>Carr, Andrew; University of Oxford, Nuffield Department of Orthopaedics, Rheumatology and Musculoskeletal Sciences<br>Beard, David; University of Oxford, Nuffield Department of Orthopaedics, Rheumatology and Musculoskeletal Sciences<br>Linton, Myles-Jay; University of Bristol, NIHR CLAHRC West<br>Rooshenas, Leila; University of Bristol, Population Health Sciences<br>Donovan, Jenny; University of Bristol, NIHR CLAHRC West<br>Hollingworth, William; University of Bristol, Population Health Sciences
**Primary Subject Heading**:	Surgery
Secondary Subject Heading:	Epidemiology, Health economics
Keywords:	subacromial decompression, shoulder surgery, England, commissioning, arthroscopy

SCHOLARONE™
Manuscripts

Title: A longitudinal study of use and cost of subacromial decompression surgery: the need for effective evaluation of surgical procedures to prevent overtreatment and wasted resources.

Authors: Tim Jones[1,2], Andrew Carr[3], David Beard[3], Myles-Jay Linton[1,2], Leila Rooshenas[2], Jenny L. Donovan[1,2], and William Hollingworth[1,2]

[1] NIHR CLAHRC West, University Hospitals Bristol NHS Foundation Trust, Bristol, BS1 2NT, UK

[2] Department of Population Health Sciences, Bristol Medical School, University of Bristol, BS8 2PS, UK

[3] Nuffield Department of Orthopaedics, Rheumatology, and Musculoskeletal Sciences (NDORMS), NIHR Biomedical Research Centre, Botnar Research Centre, University of Oxford, Oxford, UK

Corresponding author:

Tim Jones
Senior Research Associate
NIHR CLAHRC West,
9th Floor Whitefriars,
Lewin's Mead
Bristol, BS1 2NT
United Kingdom

Tel: ++44 (0)117 342 1265
Timothy.Jones@bristol.ac.uk

Word count [3,037]

Keywords:
Subacromial decompression; arthroscopy; shoulder surgery; England; commissioning

For peer review only

ABSTRACT

Objectives: To illustrate the need for better evaluation of surgical procedures, we investigated the use and cost of subacromial decompression in England over the last decade compared with other countries and explored how this related to the conduct and outcomes of randomised, placebo-controlled clinical trials.

Design: Longitudinal observational study using Hospital Episode Statistics linked to Payment by Results tariffs in England, 2007/8-2016/17.

Setting: Hospital care in England, Finland, New York State USA, Florida State USA, and Western Australia.

Participants: Patients with subacromial shoulder pain.

Interventions: Subacromial decompression.

Main outcome measures: National procedure rates, costs, and variation between clinical commissioning groups (CCGs) in England.

Results: Without robust clinical evidence, the use of subacromial decompression in England increased by 91% from 15,112 procedures (30 per 100,000 population) in 2007/8, to 28,802 procedures (52 per 100,000 population) in 2016/17, costing over £125 million per year. Rates of use of subacromial decompression are even higher internationally: Finland (131 per 100,000 in 2011), Florida State (130 per 100,000 in 2007), Western Australia (115 per 100,000 in 2013), and New York State (102 per 100,000 in 2006). Two randomised placebo-controlled trials have recently (2018) shown the procedure to be no more effective than placebo or conservative approaches. Health systems appear unable to avoid the rapid widespread use of procedures of unknown effectiveness, and methods for ceasing ineffective treatments are under-developed.

Conclusions: Without good evidence, nearly 30,000 subacromial decompression procedures have been commissioned each year in England, costing over £1 billion since 2007/8. Even higher rates of procedures are carried out in countries with less regulated health systems. High quality randomised trials need to be initiated before widespread adoption of promising operative procedures to avoid

For peer review only

over-treatment and wasted resources, and methods to prevent or desist the use of ineffective procedures need to be expedited.

For peer review only

For peer review only - http://bmjopen.bmj.com/site/about/guidelines.xhtml

ARTICLE SUMMARY

Strengths and Limitations of this Study

- Our study used a national, longitudinal dataset over a 10-year period covering all NHS secondary care providers in England, and private provision for NHS-funded patients.
- Hospital Episode Statistics are linked to hospital payments, which is a strong incentive to provide complete data, and allowed us to explore costs of subacromial decompression in England.
- We provide international comparisons of the use of subacromial decompression surgery.
- Our data are from 2007/08 onwards, so we under-estimate the amount spent on subacromial decompression prior to publication of major clinical trial results (CSAW and FIMPACT).
- There may be additional factors influencing surgery rates which we have not controlled for (e.g. private health insurance coverage).

For peer review only

For peer review only - http://bmjopen.bmj.com/site/about/guidelines.xhtml

INTRODUCTION

Health and social care services are 'straining at the seams' following increasing demand for services from an ageing population with more complex needs.[1] In England, over 200 Clinical Commissioning Groups (CCGs) have a budget to purchase health services for their local populations.[2] Hospital care currently accounts for 48.5% (£74 billion) of government health expenditure in the UK.[3] It is vital that commissioners make evidence-based decisions to maximise the effectiveness of this hospital care budget to benefit the overall health of the population.

Medicines must be licensed for use for a particular condition, requiring pharmaceutical companies to provide evidence of effectiveness from clinical trials to relevant agencies such as the Medicines and Healthcare products Regulatory Agency (MHRA) in the UK,[4] European Medicines Agency (EMA) in the EU, or the Food and Drug Administration (FDA) in the United States.[5] In the UK, the National Institute for Health and Care Excellence (NICE) also evaluates the cost-effectiveness of many medicines and does not recommend those which do not provide value. These regulatory processes have their limitations,[6] but require robust evidence for the introduction of new treatments. The quality of evidence required to introduce new surgical procedures is not as strict as for medicines,[4 5] in part because no specific product such as a drug or device is involved; it can be difficult to categorise procedures as 'new' rather than modifications; and outcomes may depend on the skill of the practitioner as well as the procedure itself.[4] Once introduced, use of procedures can spread by clinical consensus,[5] and established practice and clinical evidence often take many years to be updated.[7 8]

NHS England has recently commissioned a consultation regarding the use of 17 hospital procedures,[9] one of which is subacromial decompression for shoulder pain. Shoulder pain is common, with a lifetime prevalence of up to 66.7% [10]. Most of these cases (up to 70%) are related to rotator cuff tears or subacromial pain.[11] Subacromial pain is often considered to be caused by bony 'spurs' forming on the acromion, part of the shoulder blade, leading to inflammation in the surrounding

For peer review only - http://bmjopen.bmj.com/site/about/guidelines.xhtml

bursa and tendons.[12 13] Subacromial decompression removes the bony spur on the acromion and releases the coracohumeral ligament.[13 14] There has been a rapidly increasing use of subacromial decompression in England, with over 21,000 procedures carried out in 2009/10.[13]

Several randomised controlled trials (RCTs) since the early 1990s have compared subacromial decompression to non-operative treatment (e.g. exercise) for shoulder pain and found no evidence of effectiveness.[15-17] Two recent multi-centre RCTs including a placebo surgery arm have further questioned the effectiveness of subacromial decompression for shoulder pain.[18 19] The CSAW trial,[12 18] recruiting in England from 2012 to 2015, compared arthroscopic subacromial decompression surgery, placebo (investigational shoulder arthroscopy), and no treatment.[18] It found no difference in shoulder function after six months between the arthroscopic subacromial decompression group and the arthroscopy only (placebo) group, with a small, non-clinically significant benefit of surgery over the no treatment control. The FIMPACT trial,[19] recruiting in Finland from 2005 to 2013, compared subacromial decompression with placebo surgery and exercise therapy and echoed the results of CSAW, extending them to two years follow-up. A recent Cochrane review including CSAW, FIMPACT, and earlier RCTs, found high-certainty evidence that subacromial decompression does not improve pain, function, or health-related quality of life.[20] This seriously questions whether the resources invested in subacromial decompression represent good value for money for the NHS. As a result, a recent BMJ article made a strong recommendation against subacromial decompression surgery for chronic shoulder pain.[21]

In this study we use subacromial decompression for shoulder pain as an example to explore the relationship between evolving evidence and clinical practice for hospital procedures, including how many procedures were performed over the last 10 years and how much money was spent before RCT evidence raised questions about the procedure's value; how procedure rates compare to other countries; and how the NHS might reduce the numbers of these procedures.

For peer review only - http://bmjopen.bmj.com/site/about/guidelines.xhtml

For peer review only

METHODS

Data sources

Subacromial decompression procedures were identified using the 'admitted patient care' hospital episode statistics (HES-APC). HES is a routinely collected dataset that records all episodes of care provided to patients admitted (day case or inpatient) to NHS hospitals in England and NHS-funded patients treated in the independent sector.[22 23] Each episode in HES represents a period of care under one consultant team. Up to 20 diagnoses are recorded per episode using the International Classification of Diseases (ICD) version 10. Up to 24 clinical procedures per episode may be recorded using Office of Population, Censuses and Surveys (OPCS) (fourth revision) codes. HES also includes the Lower Super Output Area (LSOA) of residence for each patient.[24]

Identifying subacromial decompression

We extracted anonymised, individual episodes in the HES-APC (2007/8 to 2016/17) dataset. We used diagnosis and procedure codes[13] (Figure 1) to identify subacromial decompression. A small number of patients received multiple shoulder procedure episodes on the same day (0.3% of all episodes). When these were for the same procedure with the same laterality (0.25% of all episodes), we assumed coding error duplication so excluded the episodes. If a procedure was marked as bilateral (0.6%), this was counted as two procedures. We excluded patients who were not resident in England.

Estimating procedure rates

National trends over time were estimated using directly standardised procedure rates [25] (per 100,000 population), with the population of England in 2016 as our standard population. For comparison of smaller areas, we estimated indirectly standardised rates[26] per 100,000 population,

For peer review only - http://bmjopen.bmj.com/site/about/guidelines.xhtml

using the same standard population, and adjusting for deprivation and ethnicity (see Appendix A for more details).

Estimating procedure costs

Costs were estimated for each financial year by linking Healthcare Resource Group (HRG) codes for each admission in HES with the Department of Health Payment by Results National Tariffs for the appropriate financial year;[27-36] see Appendix A for more details.

International comparisons

A search of Medline and the Cumulative Index of Nursing and Allied Health (CINAHL) databases was conducted for the terms “acromioplasty” or “subacromial decompression” in conjunction with “incidence” or “prevalence” or “epidemiology”. One author (TJ) screened the results for articles including rates of subacromial decompression contemporary with our data, and further screened cited articles within included studies, as well as articles which cited included studies.

All statistical analyses were conducted using Stata/MP 14.2 for Windows and we mapped variation in procedure rates across England in 2016/17 using ArcGIS ArcMap 10.5.1 for Desktop.

Patient and public involvement

There was no patient involvement in the design or conduct of this study. Two patients involved in the CSAW trial reviewed this manuscript; they were interested by the results and the cost-focussed perspective.

For peer review only - http://bmjopen.bmj.com/site/about/guidelines.xhtml

169 **RESULTS**

170 **The use of subacromial decompression in England**

171 There were 15,112 subacromial decompression procedures (30 per 100,000 population) in 2007/8,
172 rising to 28,802 procedures (52 per 100,000 population) in 2016/17 (Figure 2), excluding those done
173 in combination with rotator cuff repair. This represents a 91% increase in the number of subacromial
174 decompressions over 10 years, with 266,692 procedures carried out in total. Most of this increase
175 took place before 2011/12, and procedure rates have slightly decreased between 2011/12 and
176 2016/17. Whilst the gender balance and age of those having shoulder surgery have remained steady
177 over the last decade, the proportion of procedures conducted as day cases, using arthroscopy,
178 and/or by independent (i.e. non-NHS) providers, have all increased (Table 1).

179

180 Table 1: Descriptive information for subacromial decompression patients, 2007/08 and 2016/17

	2007/08	*2016/17*
Procedure Count	15,112	28,802
%women	51.0	52.0
Age in years (SD)	54.94 (12.55)	54.89 (12.39)
% Arthroscopy	39.0	94.1
% Independent Providers	2.4	31.9
% Day-case	51.0	79.3

181

182

183 **The cost of subacromial decompression in England**

184 In 2016/17, the median cost of an elective admission for subacromial decompression alone was
185 £4,476. The cost of subacromial decompression in England rose from £33 million in 2007/08 to £125
186 million in 2016/17. Over the 10-year period between 2007/8 and 2016/17 just under £1.1 billion was
187 spent on subacromial decompression (excluding procedures done in combination with rotator cuff
188 repair).

189 **Variation in use of subacromial decompression in England**

For peer review only - http://bmjopen.bmj.com/site/about/guidelines.xhtml

190 In 2016/17 there was substantial variation in procedure rates between CCGs, after adjusting for age, sex, deprivation, and ethnicity profiles (Figure 3). The map demonstrates pockets of very high use (>150% of the expected rate), for example in the Reading area, Wiltshire, and East Lincolnshire. There were also areas where procedure rates were less than 50% of the expected rate, such as in Worcestershire, Gloucestershire, Swindon, and North Norfolk. In 2016/17 the ratio of procedure rates between a 'high use' CCG at the 90th percentile and a 'low use' CCG at the 10th percentile was 2.7 (95% CI: 2.2-3.4). This ratio is lower than the 2007/8 ratio of 3.6 (95% CI: 2.2-6.1), although overlapping confidence intervals suggest this may be due to chance variation; see Table 2.

198 Table 2: 90/10 percentile ratios for directly age-sex standardised rates of subacromial decompression by CCG, England, 2007/8-2016/17

Year	90th Pct	10th Pct	90/10 Ratio (95% CI*)
2007/08	53	15	3.6 (2.2-6.1)
2008/09	55	16	3.3 (2.1-5.2)
2009/10	72	27	2.6 (2.0-3.5)
2010/11	87	33	2.6 (1.9-3.6)
2011/12	89	36	2.5 (2.0-3.1)
2012/13	90	33	2.7 (2.0-3.7)
2013/14	88	34	2.6 (2.1-3.3)
2014/15	89	33	2.7 (2.0-3.7)
2015/16	81	33	2.5 (1.4-4.3)
2016/17	83	30	2.7 (2.2-3.4)

200 **Confidence intervals for rate ratios*[37]

202 **International comparison of rates of subacromial decompression**

203 Table 3 shows rates of subacromial decompression in the most recent year available from England, Finland, Florida State, New York State, and Western Australia. Rates in England were lower, often only half, that of other countries. For subacromial decompression alone, the procedure rates were lower in England (52 per 100,000 in 2016/17) than Western Australia (roughly 115 per 100,000 in 2013),[38] Florida State (130 per 100,000 in 2007),[39] and Finland (131 per 100,000 in 2011).[40]

For peer review only

Figure 2 compares trends in rates of subacromial decompression in England, Finland, Florida State, New York State, and Western Australia. The rate of increase for subacromial decompression observed in our study (x2 between 2007/8 and 2016/17) was similar to Western Australia (x2 between 2001 and 2013),[38] Finland (x2.2 between 1998 and 2007),[40] and New York State (x2.5 between 1996 and 2006),[41] but lower than Florida State (x4.4 between 2003 and 2007).[39] The use of subacromial decompression in Finland peaked in 2007 and has since been declining, at least in publicly-funded hospitals, which has been attributed to accumulating evidence that it is no more clinically effective than non-surgical alternatives.[40]

Table 3: International comparisons of age-sex-standardised rates of subacromial decompression

**Article**	**Country**	**Data Year**	**SAD Rate (per 100,000 population)**
Thorpe et al. (2016)	Western Australia	2013	~115
Paloneva et al. (2015)	Finland	2011	131
Vitale et al. (2010)	New York State	2006	102
Iyengar et al. (2014)	Florida State	2007	~130
Our Data	England	2016/17	52

Notes: Numbers for Thorpe et al (2016) and Iyengar et al. (2014) were estimated from a graph; New York State data is for subacromial decompression with/without rotator cuff repair; SAD = Subacromial Decompression[38-41]

For peer review only

DISCUSSION

Statement of principal findings

NHS England carries out nearly 30,000 subacromial decompression operations per year, at an annual cost of over £125 million. Between 2007/08 and 2016/17, 266,692 subacromial decompression procedures were carried out in England costing nearly £1.1 billion, before the addition of the CSAW and FIMPACT placebo-controlled trial results to the existing evidence prompted serious questions about the clinical benefit of the procedure. Rates of subacromial decompression alone in England have gradually declined since 2011/12, although an increasing number are carried out in combination with rotator cuff repair. There was large variation between CCGs in England, even after adjustment for demographic variables, with 'high-use' areas carrying out nearly three times as many procedures as 'low-use' areas. Procedure rates in England were notably lower than other countries, arguing against any levelling of procedure rates being due to saturation of 'demand' for shoulder surgery.

Strengths and weaknesses of the study

Our study used a national, longitudinal dataset over a 10-year period covering all NHS secondary care providers in England. Hospital Episode Statistics are administrative rather than specifically designed for research. However, HES is also linked to payments for hospitals, which is a strong incentive to provide complete data, and allowed us to produce what we believe is the first exploration of costs associated with subacromial decompression in England. Payment by results tariffs are based on average national costs and may not reflect precise costs for each hospital admission. Population denominators, and linkage to the indices of multiple deprivation and census ethnicity data, allowed us to investigate trends and variations in procedure rates standardised on age, sex, deprivation and ethnicity. HES data records patients' area of residence, so we compared procedure rates based on place of residence rather than place of treatment. There may be other

For peer review only - http://bmjopen.bmj.com/site/about/guidelines.xhtml

factors influencing rates which we have not controlled for (e.g. private health insurance coverage). HES does not record procedures which are privately funded and provided, meaning our surgery rates are an under-estimate of the population rate. We only provide cost data from 2007/08 onwards, so we under-estimate the amount spent on subacromial decompression prior to publication of the CSAW and FIMPACT trial results. International estimations of procedure rates do not use identical definitions of procedures and inclusion/exclusion criteria, but should be broadly comparable.

Implications for policymakers and clinicians

NHS England spent over £1 billion on subacromial decompression during the last 10 years without having compelling evidence of clinical effectiveness or cost-effectiveness. Rates of subacromial decompression were already rising rapidly from 2000/01 onwards.[13] It seems plausible that increasing awareness of concerns about the effectiveness of subacromial decompression surgery[15-17] and well-known recruitment to the CSAW trial tempered the rise in use of this surgery in England, otherwise more may have been spent. The CSAW trial involved 51 surgeons in 30 centres throughout the UK and was widely advertised and discussed amongst shoulder surgeons and shoulder physiotherapists. Extensive consultation was carried out by the trial team prior to and during the trial, including presentations at national meetings surveys and visits to individual surgeons and centres.[42] A similar plateau/decrease in procedures was observed in Finland after the commencement of the FIMPACT study in 2005 which involved only 3 centres in Finland (Figure 2). It is likely that awareness of a potential lack of effectiveness of subacromial decompression had been growing in the years before CSAW and FIMPACT, based on earlier trial results.[15-17] However, it took well over a decade of increasing subacromial decompression use for clinical trial groups to run high quality, low risk-of-bias, placebo-controlled studies randomising a few hundred patients (313 patients for CSAW [18] and 210 in FIMPACT[19]) to investigate its effectiveness. This delay may be due to perceived difficulties in recruiting patients to surgical trials with non-surgical comparators (e.g. UKUFF[43]), as well as known challenges of conducting surgical RCTs.[44] Methods to optimise

For peer review only - http://bmjopen.bmj.com/site/about/guidelines.xhtml

recruitment, as used in CSAW and other trials,[45] are available to support the completion of such 'difficult' trials;[46] this should not now be a barrier to rapidly initiating trials to provide robust evidence about surgical interventions before they become widespread. More time is needed to see the longer-term impact of publication of the CSAW and FIMPACT results on subacromial decompression rates, both in the UK and internationally.

The National Institute for Health and Care Excellence (NICE) requires evidence of cost-effectiveness to recommend new medicines to be paid for by the NHS. It is unclear why the bar for introducing expensive surgical procedures should be significantly lower. A balance needs to be struck between supporting innovation in surgical procedures and preventing unnecessary treatment. New initiatives such as IDEAL (Idea, Development, Exploration, Assessment, Long-term Follow-up, Improving the Quality of Research in Surgery)[47] aim to provide such a regulatory framework for introducing new interventions.

It is important that new evidence is disseminated quickly without causing inequities in access to care. NICE published an updated Clinical Knowledge Summary for shoulder pain in April 2017[48] incorporating information from a commissioning guide published by the Royal College of Surgeons.[49] This recommended a range of conservative treatments from physiotherapy to corticosteroid injections, before surgery. However, many CCGs introduced their own criteria-based policies for access to shoulder surgery (e.g. through Individual Funding Requests)[50], essentially meaning that commissioners would only pay providers for surgery under particular circumstances. These were implemented at different times and with different details, underlining the extent to which insufficient evidence may drive clinical and commissioner uncertainty,[51] and possibly leading to the wide variations shown across CCGs in our data. Where scientific evidence is applicable nationally or internationally, it would seem more efficient and appropriate to apply national policies to inform optimal use and encourage further research. There is a need to improve techniques for empirically-informed policy development in collaboration with relevant stakeholders.[52 53]

For peer review only - http://bmjopen.bmj.com/site/about/guidelines.xhtml

Despite the criticisms provided above, England has lower rates of shoulder surgery than other countries. The reasons for this are uncertain but could be due to differences in the health systems (e.g. GP gatekeeping of services), access to surgery and hospital reimbursement. Additionally, the National Institute of Health Research in England has funded major clinical trials on shoulder surgery,[18 43] as well as other procedures;[54 55] and is about to fund a further clinical trial to compare surgery with placebo surgery for partial thickness rotator cuff tears.[56] Whilst the UK's national regulatory processes are imperfect, they may provide examples to learn from. However, these processes did not sufficiently constrain the use of subacromial decompression, a procedure later found to have little clinical benefit.

There have been several other controversies regarding the lack of effectiveness of procedures which have become commonplace. One example is the use of stents to open narrowed arteries for treatment of stable angina (chest pain). Around half a million people receive stents for stable angina each year in the US and Europe,[57] but a recent (RCT) including a placebo intervention found no difference in chest pain outcomes between inserting a stent and using standard medications.[58] Another example is arthroscopy to clean out the knee joint, on which around $4 billion is spent each year in the US.[59] Recent RCTs,[60 61] including one using a placebo procedure as a comparison,[61] found no evidence of effectiveness to justify the spending. Whilst we use subacromial decompression as an example in this study, our observations are likely to apply to interventional procedures more generally.

Unanswered questions and future research

The example of subacromial decompression highlights that, in the absence of rigorous evaluation, costly interventions can proliferate over a long period of time. To maximise limited resources, it is vital that methods are developed to identify promising procedures early and commission trials to examine their value, as well as identify existing health technologies that may be ineffective, leading to over-treatment and wasting of resources.

For peer review only - http://bmjopen.bmj.com/site/about/guidelines.xhtml

There is an opportunity for a natural experiment exploring the impact of the results of the CSAW and FIMPACT trials[18 19] on the development of CCG policies, national guidelines, and clinical decision-making with surgeons and patients. It is arguable that we should now see swift reductions in the use of subacromial decompression; research studies could help enhance the transfer of knowledge from trials into clinical practice.

Conclusions

NHS England pays for nearly 30,000 shoulder subacromial decompression procedures each year at an annual cost of over £125 million, with little evidence that they are effective or cost-effective. The rates of this operation in other countries are even higher. This raises serious questions around the regulatory and professional processes governing the adoption and widespread use of surgical interventions. High quality RCTs should be funded early to examine the effectiveness and cost-effectiveness of expensive procedures using methods to optimise recruitment, and robust processes should be developed to reduce the use of ineffective procedures.

For peer review only

Author Statement

This publication is the work of the authors, who serve as guarantors for the contents of this paper. TJ contributed to study design, data cleaning, data analysis, interpretation of results and writing the manuscript. MJL contributed to study design, data cleaning, interpretation of results and writing the manuscript. AC contributed to study design, interpretation of results and writing the manuscript. DB, LR, and JD contributed to interpretation of results and writing the manuscript. WH contributed to study conceptualization, study design, interpretation of results and writing the manuscript. TJ had full access to the data in the study and takes responsibility for the integrity of the data and the accuracy of the data analysis.

Acknowledgements

The authors would like to thank two patients from the CSAW trial, Carol Brennan and Dair Farrer-Hockley, who took the time to read and comment on this manuscript.

Funding

This research was funded by the National Institute for Health Research (NIHR) Collaboration for Leadership in Applied Health Research and Care West (NIHR CLAHRC West). The views expressed in this article are those of the author(s) and not necessarily those of the NHS, the NIHR, or the Department of Health and Social Care.

Ethnical Approval

We were provided with routinely-collected Hospital Episode Statistics data under licence from NHS Digital (DARS-NIC-17875-X7K1V). The licence allows us to use the information under Section 261 of the Health and Social Care Act 2012, 2(b)(ii): “after taking into account the public interest as well as the interests of the relevant person, considers that it is appropriate for the information to be disseminated”.

Data Sharing

For peer review only - http://bmjopen.bmj.com/site/about/guidelines.xhtml

This study is based in part on data from the Hospital Episode Statistics (HES) obtained under licence (DARS-NIC-17875-X7K1V) from NHS Digital (previously the Health and Social Care Information Centre); *Copyright © 2018, re-used with the permission of The Health & Social Care Information Centre. All rights reserved*. The data are provided by patients and collected by the NHS as part of their care and support. HES data can be accessed via NHS Digital: https://digital.nhs.uk/services/data-access-request-service-dars

Transparency

The manuscript's guarantor (TJ) affirms that the manuscript is an honest, accurate, and transparent account of the study being reported; that no important aspects of the study have been omitted; and that any discrepancies from the study as originally planned have been explained.

Competing Interests

All authors have completed the ICMJE uniform disclosure form at www.icmje.org/ coi_disclosure.pdf and declare: TJ and JD had financial support from NIHR CLAHRC West for the submitted work; no financial relationships with any organisations that might have an interest in the submitted work in the previous three years; no other relationships or activities that could appear to have influenced the submitted work.

For peer review only

REFERENCES

1. Care Quality Commission. The state of health care and adult social care in England 2017 [Available from: http://www.cqc.org.uk/sites/default/files/20171017_stateofcare1617_report.pdf.
2. NHS Clinical Commissioners. About CCGs 2017 [Available from: https://www.nhscc.org/ccgs/ accessed 3rd November 2017.
3. Office for National Statistics. UK Health Accounts: 2016 2018 [Available from: https://www.ons.gov.uk/peoplepopulationandcommunity/healthandsocialcare/healthcaresystem/bulletins/ukhealthaccounts/2016.
4. Campbell B. Regulation and safe adoption of new medical devices and procedures. *Brit Med Bull* 2013;107(1):5-18. doi: 10.1093/bmb/ldt022
5. Darrow JJ. Explaining the absence of surgical procedure regulation. *Cornell Journal of Law and Public Policy* 2017;27(1):189-206.
6. Hollingworth W, Jones T, Reeves BC, et al. A longitudinal study to assess the frequency and cost of antivascular endothelial therapy, and inequalities in access, in England between 2005 and 2015. *Bmj Open* 2017;7(10) doi: 10.1136/bmjopen-2017-018289
7. Tatsioni A, Bonitsis NG, Ioannidis JPA. Persistence of contradicted claims in the literature. *Jama-J Am Med Assoc* 2007;298(21):2517-26. doi: DOI 10.1001/jama.298.21.2517
8. Jevne J. The sexy scalpel: unnecessary shoulder surgery on the rise. *Brit J Sport Med* 2015;49(16):1031-32. doi: 10.1136/bjsports-2015-094589
9. NHS England, NHS Clinical Commissioners, Academy of Medical Royal Colleges, et al. Evidence-Based Interventions: Consultation Document: NHS England, 2018.
10. Luime JJ, Koes BW, Hendriksen IJM, et al. Prevalence and incidence of shoulder pain in the general population; a systematic review. *Scand J Rheumatol* 2004;33(2):73-81. doi: 10.1080/03009740310004667
11. Mitchell C, Adebajo A, Hay E, et al. Shoulder pain: diagnosis and management in primary care. *Brit Med J* 2005;331(7525):1124-28a. doi: DOI 10.1136/bmj.331.7525.1124
12. Beard D, Rees J, Rombach I, et al. The CSAW Study (Can Shoulder Arthroscopy Work?) - a placebo-controlled surgical intervention trial assessing the clinical and cost effectiveness of arthroscopic subacromial decompression for shoulder pain: study protocol for a randomised controlled trial. *Trials* 2015;16 doi: 10.1186/s13063-015-0725-y
13. Judge A, Murphy RJ, Maxwell R, et al. Temporal trends and geographical variation in the use of subacromial decompression and rotator cuff repair of the shoulder in England. *Bone Joint J* 2014;96b(1):70-74. doi: 10.1302/0301-620x.96b1.32556
14. Chalmers PN, Romeo AA. Arthroscopic Subacromial Decompression and Acromioplasty. *Jbjs Essent Surg Tec* 2016;6(2) doi: 10.2106/JBJS.ST.O.00011
15. Brox JI, Staff PH, Ljunggren AE, et al. Arthroscopic surgery compared with supervised exercises in patients with rotator cuff disease (stage II impingement syndrome). *BMJ* 1993;307(6909):899-903. doi: 10.1136/bmj.307.6909.899 [published Online First: 1993/10/09]
16. Haahr JP, Ostergaard S, Dalsgaard J, et al. Exercises versus arthroscopic decompression in patients with subacromial impingement: a randomised, controlled study in 90 cases with a one year follow up. *Ann Rheum Dis* 2005;64(5):760-4. doi: 10.1136/ard.2004.021188 [published Online First: 2005/04/19]
17. Ketola S, Lehtinen J, Arnala I, et al. Does arthroscopic acromioplasty provide any additional value in the treatment of shoulder impingement syndrome?: a two-year randomised controlled trial. *J Bone Joint Surg Br* 2009;91(10):1326-34. doi: 10.1302/0301-620X.91B10.22094 [published Online First: 2009/10/02]
18. Beard DJ, Rees JL, Cook JA, et al. Arthroscopic subacromial decompression for subacromial shoulder pain (CSAW): a multicentre, pragmatic, parallel group, placebo-controlled, three-group, randomised surgical trial. *The Lancet* 2017 doi: 10.1016/s0140-6736(17)32457-1

For peer review only - http://bmjopen.bmj.com/site/about/guidelines.xhtml

19. Paavola M, Malmivaara A, Taimela S, et al. Subacromial decompression versus diagnostic arthroscopy for shoulder impingement: randomised, placebo surgery controlled clinical trial. *Bmj-Brit Med J* 2018;362 doi: 10.1136/bmj.k2860
20. Karjalainen TV, Jain NB, Page CM, et al. Subacromial decompression surgery for rotator cuff disease. *Cochrane Db Syst Rev* 2019(1) doi: 10.1002/14651858.CD005619.pub3
21. Vandvik PO, Lähdeoja T, Ardern C, et al. Subacromial decompression surgery for adults with shoulder pain: a clinical practice guideline. *BMJ* 2019;364:l294. doi: 10.1136/bmj.l294
22. Health and Social Care Information Centre. Hospital Episode Statistics (HES) Analysis Guide, 2015.
23. Herbert A, Wijlaars L, Zylbersztejn A, et al. Data Resource Profile: Hospital Episode Statistics Admitted Patient Care (HES APC). *Int J Epidemiol* 2017;46(4):1093-+. doi: 10.1093/ije/dyx015
24. NHS Digital. Lower Layer Super Output Area [Available from: https://www.datadictionary.nhs.uk/data_dictionary/nhs_business_definitions/l/lower_layer_super_output_area_de.asp?shownav=1 accessed 14 May 2018.
25. Standardization: a classic epidemiological method for the comparison of rates. *Epidemiol Bull* 2002;23(3):9-12. [published Online First: 2003/03/01]
26. Anon. Standardization: a classic epidemiological method for the comparison of rates. *Epidemiological Bulletin* 2002;23(3):9-12.
27. Department of Health. Payment by Results National Tariffs 2016/17 2016 [Available from: https://www.gov.uk/government/publications/nhs-national-tariff-payment-system-201617 accessed 30 November 2017.
28. Department of Health. Payment by Results Enhanced Tariff Option 2015/16 2015 [Available from: http://webarchive.nationalarchives.gov.uk/20161103233943/https://www.england.nhs.uk/resources/pay-syst/tariff-guide/ accessed 30 November 2017.
29. Department of Health. Payment by Results National Tariffs 2014/15 2014 [Available from: https://www.gov.uk/government/publications/national-tariff-payment-system-2014-to-2015 accessed 30 November 2017.
30. Department of Health. Payment by Results National Tariffs 2013/14 2013 [Available from: https://www.gov.uk/government/publications/payment-by-results-pbr-operational-guidance-and-tariffs accessed 30 November 2017.
31. Department of Health. Payment by Results National Tariffs 2012/13 2012 [Available from: https://www.gov.uk/government/publications/confirmation-of-payment-by-results-pbr-arrangements-for-2012-13 accessed 30 November 2017.
32. Department of Health. Payment by Results National Tariffs 2011/12 2011 [Available from: http://webarchive.nationalarchives.gov.uk/20130507170152/https://www.gov.uk/government/publications/confirmation-of-payment-by-results-pbr-arrangements- for-2011-12 accessed 30 November 2017.
33. Department of Health. Payment by Results National Tariffs 2010/11 2010 [Available from: https://data.gov.uk/dataset/payment-by-results-2010-11-national-tariff-information accessed 30 November 2017.
34. Department of Health. Payment by Results National Tariffs 2009/10 2009 [Available from: http://webarchive.nationalarchives.gov.uk/+/http://www.dh.gov.uk/en/Publicationsandstatistics/Publications/PublicationsPolicyAndGuidance/DH_094091 accessed 30 November 2017.
35. Department of Health. Payment by Results National Tariffs 2008/09 2008 [Available from: http://webarchive.nationalarchives.gov.uk/+/http://www.dh.gov.uk/en/Managingyourorganisation/Financeandplanning/NHSFinancialReforms/DH_081238 accessed 30 November 2017.
36. Department of Health. Payment by Results National Tariffs 2007/08 2007 [Available from: http://webarchive.nationalarchives.gov.uk/+/http://www.dh.gov.uk/en/Publicationsandstat

For peer review only

istics/Publications/PublicationsPolicyAndGuidance/DH_062914 accessed 30 November 2017.
37. Kirkwood BR, Sterne JA. Rate Ratios. Essential Medical Statistics. Oxford, UK: Blackwell Science Ltd 2003:241-42.
38. Thorpe A, Hurworth M, O'Sullivan P, et al. Rising trends in surgery for rotator cuff disease in Western Australia. *Anz J Surg* 2016;86(10):801-04. doi: 10.1111/ans.13691
39. Iyengar JJ, Samagh SP, Schairer W, et al. Current Trends in Rotator Cuff Repair: Surgical Technique, Setting, and Cost. *Arthroscopy* 2014;30(3):284-88. doi: 10.1016/j.arthro.2013.11.018
40. Paloneva J, Lepola V, Karppinen J, et al. Declining incidence of acromioplasty in Finland. *Acta Orthop* 2015;86(2):220-24. doi: 10.3109/17453674.2014.977703
41. Vitale MA, Arons RR, Hurwitz S, et al. The Rising Incidence of Acromioplasty. *J Bone Joint Surg Am* 2010;92a(9):1842-50. doi: 10.2106/Jbjs.I.01003
42. Wartolowska K, Beard DJ, Carr AJ. Attitudes and Beliefs about Placebo Surgery among Orthopedic Shoulder Surgeons in the United Kingdom. *Plos One* 2014;9(3) doi: 10.1371/journal.pone.0091699
43. Carr A, Cooper C, Campbell MK, et al. Effectiveness of open and arthroscopic rotator cuff repair (UKUFF) A RANDOMISED CONTROLLED TRIAL. *Bone Joint J* 2017;99b(1):107-15. doi: 10.1302/0301-620x.99b1.Bjj-2016-0424.R1
44. Cook JA, Ramsay CR, Norrie J. Recruitment to publicly funded trials - Are surgical trials really different? *Contemp Clin Trials* 2008;29(5):631-34. doi: 10.1016/j.cct.2008.02.005
45. Rooshenas L, Scott LJ, Blazeby JM, et al. The Quintet Recruitment Intervention supported five randomized trials to recruit to target: a mixed-methods evaluation 2018 [Available from: https://doi.org/10.1016/j.jclinepi.2018.10.004.
46. Donovan JL, Rooshenas L, Jepson M, et al. Optimising recruitment and informed consent in randomised controlled trials: the development and implementation of the Quintet Recruitment Intervention (QRI). *Trials* 2016;17 doi: 10.1186/s13063-016-1391-4
47. Hirst A, Philippou Y, Blazeby J, et al. No Surgical Innovation Without Evaluation: Evolution and Further Development of the IDEAL Framework and Recommendations. *Annals of Surgery* 2018 doi: 10.1097/SLA.0000000000002794
48. National Institute for Health and Care Excellence. Shoulder Pain, 2017.
49. The Royal College of Surgeons of England. Commissioning guide: Subacromial shoulder pain, 2014.
50. Iacobucci G. Pressure on NHS finances drives new wave of postcode rationing. *Bmj-Brit Med J* 2017;358 doi: 10.1136/bmj.j3190
51. Wennberg JE, Barnes BA, Zubkoff M. Professional Uncertainty and the Problem of Supplier-Induced Demand. *Soc Sci Med* 1982;16(7):811-24. doi: Doi 10.1016/0277-9536(82)90234-9
52. Hollingworth W, Rooshenas L, Busby J, et al. Using clinical practice variations as a method for commissioners and clinicians to identify and prioritise opportunities for disinvestment in health care: a cross-sectional study, systematic reviews and qualitative study. *Health Services and Delivery Research* 2015 doi: 10.3310/hsdr03130
53. Rooshenas L, Owen-Smith A, Hollingworth W, et al. "I won't call it rationing ...": An ethnographic study of healthcare disinvestment in theory and practice. *Soc Sci Med* 2015;128:273-81. doi: 10.1016/j.socscimed.2015.01.020
54. Hamdy FC, Donovan JL, Lane JA, et al. 10-Year Outcomes after Monitoring, Surgery, or Radiotherapy for Localized Prostate Cancer. *New Engl J Med* 2016;375(15):1415-24. doi: 10.1056/NEJMoa1606220
55. Rubie I, Haighton C, O'Hara J, et al. The NAtional randomised controlled Trial of Tonsillectomy IN Adults (NATTINA): a clinical and cost-effectiveness study: study protocol for a randomised control trial. *Trials* 2015;16 doi: 10.1186/s13063-015-0768-0

For peer review only

For peer review only - http://bmjopen.bmj.com/site/about/guidelines.xhtml

56. National Institute for Health Research. 18/109 Management of Partial Thickness Rotator-Cuff Tears 2018 [Available from: https://www.nihr.ac.uk/funding-and-support/funding-opportunities/18109-management-of-partial-thickness-rotator-cuff-tears/89052018.
57. Belluz J. Thousands of heart patients get stents that may do more harm than good: Vox; 2017 [Available from: https://www.vox.com/science-and-health/2017/11/3/16599072/stent-chest-pain-treatment-angina-not-effective.
58. Al-Lamee R, Thompson D, Dehbi HM, et al. Percutaneous coronary intervention in stable angina (ORBITA): a double-blind, randomised controlled trial. *Lancet* 2018;391(10115):31-40. doi: 10.1016/S0140-6736(17)32714-9
59. Epstein D. When evidence says no, but doctors say yes: ProPublica; 2017 [Available from: https://web.archive.org/web/20170316151510/https://www.propublica.org/article/when-evidence-says-no-but-doctors-say-yes2018.
60. Katz JN, Brophy RH, Chaisson CE, et al. Surgery versus Physical Therapy for a Meniscal Tear and Osteoarthritis. *New Engl J Med* 2013;368(18):1675-84. doi: 10.1056/NEJMoa1301408
61. Sihvonen R, Paavola M, Malmivaara A, et al. Arthroscopic Partial Meniscectomy versus Sham Surgery for a Degenerative Meniscal Tear. *New Engl J Med* 2013;369(26):2515-24. doi: 10.1056/NEJMoa1305189

For peer review only

FIGURE CAPTIONS

Figure 1. ICD-10 and OPCS-4 codes used to define subacromial decompression[13]

Figure 2. Directly standardised rates (per 100,000 people) of subacromial decompression in England, Finland, New York State USA, Florida State USA, and Western Australia

Notes for Figure 2. England data prior to 2007 is taken from Judge et al.[13]; New York State data is for subacromial decompression with or without rotator cuff repair, whilst data for Florida State, Finland and Western Australia is for subacromial decompression alone [38-41]

Figure 3. Indirectly standardised rates of subacromial decompression by CCG in England, 2016/17

For peer review only

For peer review only - http://bmjopen.bmj.com/site/about/guidelines.xhtml

<table>
<tr><th>Any of these diagnosis codes in any position</th><th rowspan="5">In combination with…</th><th>Any of these procedure codes in any position</th></tr>
<tr><td>M75.1 Rotator cuff syndrome</td><td>W84.8 Other specified therapeutic endoscopic operations on other joint structure</td></tr>
<tr><td>M75.3 Calcific tendinitis of shoulder</td><td>Y52.8 Other specified approach to organ through other opening</td></tr>
<tr><td>M75.4 Impingement syndrome of shoulder</td><td>Y76.7 Arthroscopic approach to joint</td></tr>
<tr><td>M75.5 Bursitis of shoulder</td><td>W84.4 Endoscopic decompression of joint</td></tr>
</table>

OR

*This **procedure** code in any position*
O29.1 Subacromial decompression

Figure 1. ICD-10 and OPCS-4 codes used to define subacromial decompression[13]

102x65mm (300 x 300 DPI)

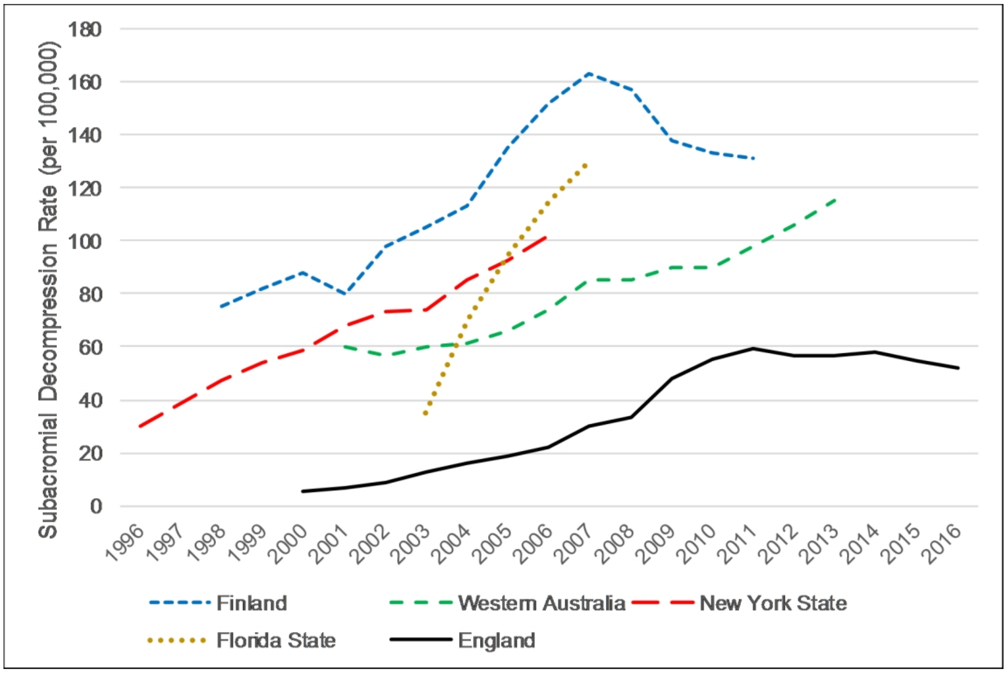

Figure 2. Directly standardised rates (per 100,000 people) of subacromial decompression in England, Finland, New York State USA, Florida State USA, and Western AustraliaNotes for Figure 2. England data prior to 2007 is taken from Judge et al.13; New York State data is for subacromial decompression with or without rotator cuff repair, whilst data for Florida State, Finland and Western Australia is for subacromial decompression alone [34-37]

150x99mm (300 x 300 DPI)

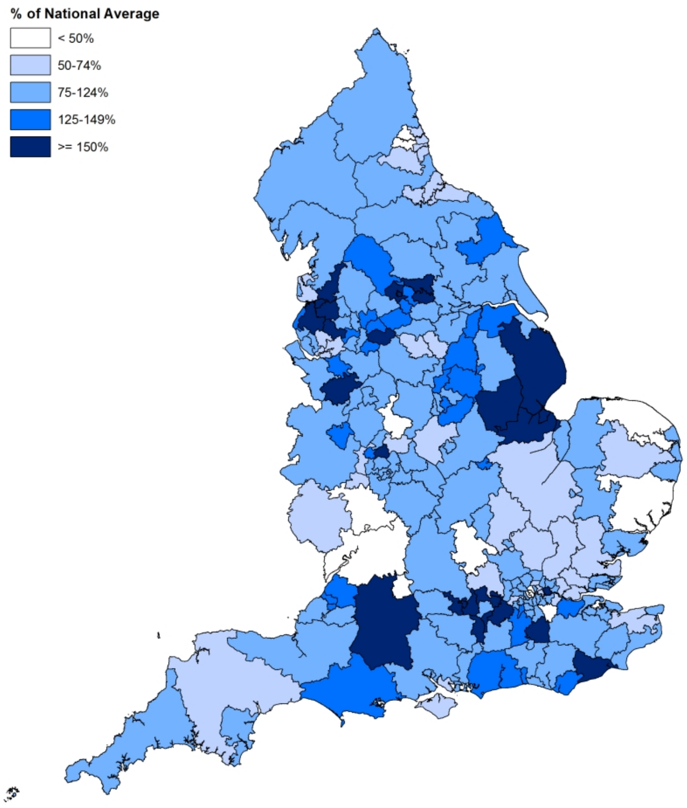

Figure 3. Indirectly standardised rates of subacromial decompression by CCG in England, 2016/17

210x296mm (300 x 300 DPI)

APPENDIX A: METHODOLOGICAL DETAILS

Estimating Procedure Rates

National trends over time were estimated using directly standardised procedure rates[1] (per 100,000 population), with the population of England in 2016 as our standard population. We first summed the number of shoulder procedures, grouped by sex, quintiles of age, and financial year. These procedure counts were used to calculate annual age-sex-specific rates, by dividing by the appropriate age-sex-specific mid-year populations of England[2] (e.g. for the 2012/13 financial year, the mid-2012 populations were used). We weighted the annual age-sex-specific rates according to the population distribution of England in 2016, to produce directly standardised rates for each year. The standardised rates for 2016/17 are the same as the crude rates.

For comparison of smaller areas, we estimated indirectly standardised rates[3] per 100,000 population. We first calculated age-sex-specific rates for England in 2016/17, then multiplied these rates by the age-sex-specific population for the area of interest[2 4 5] (e.g. CCG) and summed the results. This produced the expected number of patients and procedures for that area, if it were to have the same age-sex-specific rates as England. The expected number was then compared to the observed number of patients and procedures for that area. A Poisson regression model was fitted to the observed counts for each year, with the expected counts as an offset and socio-economic deprivation (using the overall score from the English Indices of Multiple Deprivation[6]) and ethnicity (% white British[7]) as predictive factors. The model was then used to predict new expected counts for each area based on deprivation and ethnicity, and form indirectly standardised procedure ratios (observed / expected).

Estimating Procedure Costs

Costs were estimated for each financial year by linking HRG codes for each admission in HES with the Department of Health Payment by Results National Tariffs for the appropriate financial year.[8-17] Enhanced Tariff Option (ETO) tariffs were applied for 2015/16 as, following a dispute, 88% of providers agreed to use ETO tariffs for that financial year.[18] The National Tariffs provide costs for day cases and longer stays, for both elective and non-elective admissions. They also provide additional daily costs for admissions that go above a threshold number of days (termed excess bed days), which varies for different types of admission. To calculate the cost of admission, we excluded admissions without a discharge date (used to calculate number of bed days) or without a HRG code that matched to the National Tariffs (0.7% excluded). We then applied the relevant national tariff or alternatively the best practice tariff where applicable (only for HRG code HB62C under specified circumstances) and added excess bed day costs (if there were any). Following this, the special service top-up for orthopaedic procedures was applied for each year.

References

1. Standardization: a classic epidemiological method for the comparison of rates. *Epidemiol Bull* 2002;23(3):9-12. [published Online First: 2003/03/01]
2. Office for National Statistics. Lower Super Output Area Mid-Year Population Estimates [Available from: https://www.ons.gov.uk/peoplepopulationandcommunity/populationandmigration/populationestimates/datasets/lowersuperoutputareamidyearpopulationestimates accessed 30 November 2017.
3. Anon. Standardization: a classic epidemiological method for the comparison of rates. *Epidemiological Bulletin* 2002;23(3):9-12.
4. Office for National Statistics. Lower Layer Super Output Area (2011) to Clinical Commissioning Group to Local Authority District (July 2015) Lookup in England: Office for National Statistics; [Available from: http://ons.maps.arcgis.com/home/item.html?id=7de88cd2369d4006925de49e8f3f2e272016.
5. Office for National Statistics. Output Area to Lower Layer Super Output Area to Middle Layer Super Output Area to Local Authority District (December 2011) Lookup in England and Wales [Available from: http://ons.maps.arcgis.com/home/item.html?id=baf6566ca08949c6bbd61ff81d9526da2016.

For peer review only - http://bmjopen.bmj.com/site/about/guidelines.xhtml

6. Government DoCaL. English Indices of Deprivation 2015 [Available from: https://www.gov.uk/government/statistics/english-indices-of-deprivation-20152016.
7. Nomis; Office for National Statistics. Ethnic group of the usual resident population of England and Wales as at census day, 27 March 2011 [Available from: https://www.nomisweb.co.uk/census/2011/ks201ew2016.
8. Department of Health. Payment by Results National Tariffs 2016/17 2016 [Available from: https://www.gov.uk/government/publications/nhs-national-tariff-payment-system-201617 accessed 30 November 2017.
9. Department of Health. Payment by Results Enhanced Tariff Option 2015/16 2015 [Available from: http://webarchive.nationalarchives.gov.uk/20161103233943/https://www.england.nhs.uk/resources/pay-syst/tariff-guide/ accessed 30 November 2017.
10. Department of Health. Payment by Results National Tariffs 2014/15 2014 [Available from: https://www.gov.uk/government/publications/national-tariff-payment-system-2014-to-2015 accessed 30 November 2017.
11. Department of Health. Payment by Results National Tariffs 2013/14 2013 [Available from: https://www.gov.uk/government/publications/payment-by-results-pbr-operational-guidance-and-tariffs accessed 30 November 2017.
12. Department of Health. Payment by Results National Tariffs 2012/13 2012 [Available from: https://www.gov.uk/government/publications/confirmation-of-payment-by-results-pbr-arrangements-for-2012-13 accessed 30 November 2017.
13. Department of Health. Payment by Results National Tariffs 2011/12 2011 [Available from: http://webarchive.nationalarchives.gov.uk/20130507170152/https://www.gov.uk/government/publications/confirmation-of-payment-by-results-pbr-arrangements- for-2011-12 accessed 30 November 2017.
14. Department of Health. Payment by Results National Tariffs 2010/11 2010 [Available from: https://data.gov.uk/dataset/payment-by-results-2010-11-national-tariff-information accessed 30 November 2017.
15. Department of Health. Payment by Results National Tariffs 2009/10 2009 [Available from: http://webarchive.nationalarchives.gov.uk/+/http://www.dh.gov.uk/en/Publicationsandstatistics/Publications/PublicationsPolicyAndGuidance/DH_094091 accessed 30 November 2017.
16. Department of Health. Payment by Results National Tariffs 2008/09 2008 [Available from: http://webarchive.nationalarchives.gov.uk/+/http://www.dh.gov.uk/en/Managingyourorganisation/Financeandplanning/NHSFinancialReforms/DH_081238 accessed 30 November 2017.
17. Department of Health. Payment by Results National Tariffs 2007/08 2007 [Available from: http://webarchive.nationalarchives.gov.uk/+/http://www.dh.gov.uk/en/Publicationsandstatistics/Publications/PublicationsPolicyAndGuidance/DH_062914 accessed 30 November 2017.
18. Grasic K, Mason AR, Street A. Paying for the quantity and quality of hospital care: the foundations and evolution of payment policy in England. *Health Econ Rev* 2015;5 doi: 10.1186/s13561-015-0050-x

For peer review only

The RECORD statement – checklist of items, extended from the STROBE statement, that should be reported in observational studies using routinely collected health data.

	Item No.	STROBE items	Location in manuscript where items are reported	RECORD items	Location in manuscript where items are reported
**Title and abstract**					
	1	(a) Indicate the study's design with a commonly used term in the title or the abstract (b) Provide in the abstract an informative and balanced summary of what was done and what was found		RECORD 1.1: The type of data used should be specified in the title or abstract. When possible, the name of the databases used should be included.<br><br>RECORD 1.2: If applicable, the geographic region and timeframe within which the study took place should be reported in the title or abstract.<br><br>RECORD 1.3: If linkage between databases was conducted for the study, this should be clearly stated in the title or abstract.	40, 41<br><br>37, 40, 41, 42<br><br>40, 41
**Introduction**					
Background rationale	2	Explain the scientific background and rationale for the investigation being reported			77-115
Objectives	3	State specific objectives, including any prespecified hypotheses			116-120
**Methods**					
Study Design	4	Present key elements of study design early in the paper			126-163
Setting	5	Describe the setting, locations, and relevant dates, including periods of recruitment, exposure, follow-up, and data collection			127-142

For peer review only

Participants	6	*(a) Cohort study* - Give the eligibility criteria, and the sources and methods of selection of participants. Describe methods of follow-up<br>*Case-control study* - Give the eligibility criteria, and the sources and methods of case ascertainment and control selection. Give the rationale for the choice of cases and controls<br>*Cross-sectional study* - Give the eligibility criteria, and the sources and methods of selection of participants<br><br>*(b) Cohort study* - For matched studies, give matching criteria and number of exposed and unexposed<br>*Case-control study* - For matched studies, give matching criteria and the number of controls per case		RECORD 6.1: The methods of study population selection (such as codes or algorithms used to identify subjects) should be listed in detail. If this is not possible, an explanation should be provided.<br><br>RECORD 6.2: Any validation studies of the codes or algorithms used to select the population should be referenced. If validation was conducted for this study and not published elsewhere, detailed methods and results should be provided.<br><br>RECORD 6.3: If the study involved linkage of databases, consider use of a flow diagram or other graphical display to demonstrate the data linkage process, including the number of individuals with linked data at each stage.	136-142 and Figure 1<br><br>Previous paper using same codes referenced: 137<br><br>Linked to payment-by-results tariffs by HRG code (150-152), more of a lookup table than a core linkage; also Appendix A
Variables	7	Clearly define all outcomes, exposures, predictors, potential confounders, and effect modifiers. Give diagnostic criteria, if applicable.		RECORD 7.1: A complete list of codes and algorithms used to classify exposures, outcomes, confounders, and effect modifiers should be provided. If these cannot be reported, an explanation should be provided.	136-142 and Figure 1
Data sources/ measurement	8	For each variable of interest, give sources of data and details of methods of assessment (measurement).<br>Describe comparability of assessment methods if there is more than one group			136-152

For peer review only - http://bmjopen.bmj.com/site/about/guidelines.xhtml

Bias	9	Describe any efforts to address potential sources of bias			136-148; standardisation
Study size	10	Explain how the study size was arrived at			136-142, 170-173
Quantitative variables	11	Explain how quantitative variables were handled in the analyses. If applicable, describe which groupings were chosen, and why			143-160
Statistical methods	12	(a) Describe all statistical methods, including those used to control for confounding<br>(b) Describe any methods used to examine subgroups and interactions<br>(c) Explain how missing data were addressed<br>(d) *Cohort study* - If applicable, explain how loss to follow-up was addressed<br>*Case-control study* - If applicable, explain how matching of cases and controls was addressed<br>*Cross-sectional study* - If applicable, describe analytical methods taking account of sampling strategy<br>(e) Describe any sensitivity analyses			143-160
Data access and cleaning methods		..		RECORD 12.1: Authors should describe the extent to which the investigators had access to the database population used to create the study population.	366-370

For peer review only

				RECORD 12.2: Authors should provide information on the data cleaning methods used in the study.	136-142
Linkage		..		RECORD 12.3: State whether the study included person-level, institutional-level, or other data linkage across two or more databases. The methods of linkage and methods of linkage quality evaluation should be provided.	Linked to payment-by-results tariffs by HRG code (150-152), more of a lookup table than a core linkage; also Appendix A
**Results**					
Participants	13	(a) Report the numbers of individuals at each stage of the study (*e.g.*, numbers potentially eligible, examined for eligibility, confirmed eligible, included in the study, completing follow-up, and analysed)<br>(b) Give reasons for non-participation at each stage.<br>(c) Consider use of a flow diagram		RECORD 13.1: Describe in detail the selection of the persons included in the study (*i.e.,* study population selection) including filtering based on data quality, data availability and linkage. The selection of included persons can be described in the text and/or by means of the study flow diagram.	170-179, Table 1, Appendix A
Descriptive data	14	(a) Give characteristics of study participants (*e.g.*, demographic, clinical, social) and information on exposures and potential confounders<br>(b) Indicate the number of participants with missing data for each variable of interest<br>(c) *Cohort study* - summarise follow-up time (*e.g.*, average and total amount)			Table 1, 181-182
Outcome data	15	*Cohort study* - Report numbers of outcome events or summary measures over time			170-179

For peer review only

		Case-control study - Report numbers in each exposure category, or summary measures of exposure<br>*Cross-sectional study* - Report numbers of outcome events or summary measures			
Main results	16	(a) Give unadjusted estimates and, if applicable, confounder-adjusted estimates and their precision (e.g., 95% confidence interval). Make clear which confounders were adjusted for and why they were included<br>(b) Report category boundaries when continuous variables were categorized<br>(c) If relevant, consider translating estimates of relative risk into absolute risk for a meaningful time period			170-200
Other analyses	17	Report other analyses done—e.g., analyses of subgroups and interactions, and sensitivity analyses			204-218
**Discussion**					
Key results	18	Summarise key results with reference to study objectives			231-240
Limitations	19	Discuss limitations of the study, taking into account sources of potential bias or imprecision. Discuss both direction and magnitude of any potential bias		RECORD 19.1: Discuss the implications of using data that were not created or collected to answer the specific research question(s). Include discussion of misclassification bias, unmeasured confounding, missing data, and changing eligibility over time, as they pertain to the study being reported.	242-257

For peer review only

Interpretation	20	Give a cautious overall interpretation of results considering objectives, limitations, multiplicity of analyses, results from similar studies, and other relevant evidence			259-321
Generalisability	21	Discuss the generalisability (external validity) of the study results			319-321
**Other Information**					
Funding	22	Give the source of funding and the role of the funders for the present study and, if applicable, for the original study on which the present article is based			361-364
Accessibility of protocol, raw data, and programming code		..		RECORD 22.1: Authors should provide information on how to access any supplemental information such as the study protocol, raw data, or programming code.	372-377

*Reference: Benchimol EI, Smeeth L, Guttmann A, Harron K, Moher D, Petersen I, Sørensen HT, von Elm E, Langan SM, the RECORD Working Committee. The REporting of studies Conducted using Observational Routinely-collected health Data (RECORD) Statement. *PLoS Medicine* 2015; in press.

*Checklist is protected under Creative Commons Attribution (CC BY) license.

For peer review only
